# Transmission Intensity and Drug Resistance in Malaria Population Dynamics: Implications for Climate Change

**DOI:** 10.1371/journal.pone.0013588

**Published:** 2010-10-26

**Authors:** Yael Artzy-Randrup, David Alonso, Mercedes Pascual

**Affiliations:** 1 Howard Hughes Medical Institute, Department of Ecology and Evolutionary Biology, University of Michigan, Ann Arbor, Michigan, United States of America; 2 Community and Conservation Ecology Group, Center for Ecological and Evolutionary Studies, University of Groningen, Groningen, The Netherlands; University of Leeds, United Kingdom

## Abstract

Although the spread of drug resistance and the influence of climate change on malaria are most often considered separately, these factors have the potential to interact through altered levels of transmission intensity. The influence of transmission intensity on the evolution of drug resistance has been addressed in theoretical studies from a population genetics' perspective; less is known however on how epidemiological dynamics at the population level modulates this influence. We ask from a theoretical perspective, whether population dynamics can explain non-trivial, non-monotonic, patterns of treatment failure with transmission intensity, and, if so, under what conditions. We then address the implications of warmer temperatures in an East African highland, where, as in other similar regions at the altitudinal edge of malaria's distribution, there has been a pronounced increase of cases from the 1970s to the 1990s. Our theoretical analyses, with a transmission model that includes different levels of immunity, demonstrate that an increase in transmission beyond a threshold can lead to a decrease in drug resistance, as previously shown [1], but that a second threshold may occur and lead to the re-establishment of drug resistance. Estimates of the increase in transmission intensity from the 1970s to the 1990s for the Kenyan time series, obtained by fitting the two-stage version of the model with an explicit representation of vector dynamics, suggest that warmer temperatures are likely to have moved the system towards the first threshold, and in so doing, to have promoted the faster spread of drug resistance. Climate change and drug resistance can interact and need not be considered as alternative explanations for trends in disease incidence in this region. Non-monotonic patterns of treatment failure with transmission intensity similar to those described as the ‘valley phenomenon’ for Uganda can result from epidemiological dynamics but under poorly understood assumptions.

## Introduction

Evolutionary change is one main challenge to the understanding and prediction of climate change impacts on biological systems. Environmental and evolutionary drivers of biological change are likely to interact in ways poorly understood. One area where this interaction has the potential to act on relatively fast time scales is the dynamics of vector-transmitted diseases. These diseases, such as malaria, are especially sensitive to changes in environmental conditions. Climate change expressed through changes in temperature and precipitation influences habitat suitability and can potentially shift the geographical range of malaria. Warmer temperatures accelerate physiological processes of the mosquito vector, leading to increased activity such as biting rate, growth, development and reproduction. Extreme temperatures may also decrease survivorship of vectors, leading to a convex relationship between temperature and mosquito performance. Physiological processes of parasite development within the vector are also affected by temperature levels in a nonlinear way [2]–[5]. In particular, temperature plays a key limiting role on malaria at the edge of the altitudinal distribution of the disease, in highland regions, where the parasite is not likely to complete development during the lifetime of its vector.

Climate change and drug resistance have been typically addressed as independent drivers of malaria trends, and have been considered as alternative explanations for the exacerbation of the disease in East African highlands [6]–[8], [4]. This was sensible when analyses of temperature records showed no evidence for a trend in climate variables [6]; more recent re-analyses of these records now indicate that temperature trends are present [8], [9], also in local temperatures [9], raising the possibility of synergistic interactions between warmer temperatures and drug resistance, in the larger context of pathogen evolution under changing environmental conditions. In one direction, the non-independence of these factors is trivial: we clearly do not expect to see any increase in transmission and prevalence when drugs are available and effective. Thus, the failure of treatment is a pre-requisite for an effect of climate change in areas with access to treatment by effective drugs. There are excellent examples of comparisons between locations with different drug policies, including the introduction of new, effective, drugs with clear contrasts in the occurrence of epidemics (e.g., [7]. It is the opposite direction that interests us here: the influence of higher transmission, driven by climate change, on the spread of drug resistance.

Numerous modelling studies have addressed the interplay of transmission intensity and drug resistance from a population genetics perspective (see: [10]–[13]). Less attention has been given to how the population dynamics of the disease modulates this interaction (e.g., [1], [14], [15]). Among these studies, two recent epidemiological models propose that low, rather than high, transmission would favor the spread of drug resistance [1], [14]. These studies also emphasize the importance of immunity considerations. In particular, Klein *et al.* (2008) show that clinical immunity can act as a refuge for the wild type, non-resistant, strain, and in so doing, oppose the emergence of drug resistance at high transmission levels for which population structure shifts towards providing such a refuge. This mechanism was proposed as an explanation for the empirical observation of the *de novo* evolution of drug resistance in areas of low transmission intensity. Throughout this paper, our focus is not on the *de novo* evolution of drug resistance, but on the invasion and spread of resistance when introduced in a population (for recent results on the former, see [12], [13]). While the former is a rare (albeit important) event, the latter affects the prevalence of drug resistance in large regions of the globe [16].

We specifically examine the role of immunity in the evolution of drug resistance further, with a particular focus on conditions that influence the existence of thresholds in the selective advantage of drug resistance as a function of transmission intensity. We extend the analyses of Klein *et al.* (2008) in three ways. First, we consider an epidemiological model that integrates multiple levels of immunity within a population of hosts. The approach we use stems from the idea that transmission intensity structures a local community into a series of immunity classes, such that under different climatic settings, the distribution of hosts between immunity classes varies. The model is constructed as a multi-stage SIS model with different levels of immunity as a function of exposure, and is an extension of the previously proposed two-stage model [1]. Analytical analysis and simulations demonstrate that at low transmission settings changes increase in transmission acts to encourage the spread of drug resistance, and that further increase in transmission beyond a threshold can lead to a decrease in drug resistance, as previously shown by Klein *et al.* (2008), but that a second threshold can occur, where further increases in transmission lead to the re-establishment of drug resistance. This work identifies the main assumptions required for the existence of both thresholds, and therefore, for the existence of a non-monotonic pattern in which the fitness of the drug resistance parasite first increases, then decreases, and increases again. A second modification of the original two-stage model is also theoretical, introducing in a phenomenological way, the observation that parasite antigenic diversity increases with transmission. This is shown to move the first threshold to higher values of transmission, making it a moving target and hence extending the range of transmission intensities that increase the fitness of the drug resistant parasite.

A third extension adds to the two-stage model for transmission in humans, a mosquito submodel for the population dynamics of the vector as a function of observed temperatures and rainfall. With this model, we seek to determine where the system lies, relative to the first threshold, in an East African highland as temperatures increase from the 1970s to the 1990s. Increases in transmission intensity below this point would have promoted the spread of drug resistance. To this end, we consider a time series of malaria cases from a tea plantation in the Kenyan highlands that has been at the center of retrospective malaria studies [5], [7], [8], [17] Although it is in these regions at the edge of the geographical distribution of the disease, where altitude limits transmission, that effects of climate change are expected to be most apparent, the underlying and dominant causes for larger and more frequent epidemics from the 1970s to the 1990s are not yet clear [6]–[8], [18], [19]. Climate change is one among several factors that have been proposed as the driving forces responsible for the exacerbation of the disease, including the spread of anti-malarial drug resistance, declining health services provision, reduced vector control and increased host abundance [6], [7]. Although chloroquine (CQ) resistance first appeared in Kenya in the late 1970s, in 1985 parasites were still found to be sensitive to the drug in a highland district adjacent to this tea plantation [20]. By 1996, however, the drug was unable to clear approximately half of the clinical infections in children in the Kericho hospital [21]. Interestingly, the emergence of drug resistance in the Kenyan highlands overlaps in timing with the rise in temperatures over the past three decades [8], [9], making it difficult to consider these drivers independently. Our results suggest that the observed temperature trend has been sufficient to alter the epidemiological structure of the population, and that in doing so, this trend would not have acted independently from drug resistance.

We end with implications of our findings in relation to empirical patterns, including the ‘valley phenomenon’ described for CQ in the Uganda highlands, where treatment failure was found to be higher in areas of low and high transmission, and at it's lowest at intermediate transmission areas [22]–[24]. We also discuss epidemiological assumptions identified by our analyses, and open questions on key aspects of parasite diversity of relevance to these assumptions that are still poorly understood, at the interface of molecular studies, within-host dynamics, and between-host transmission.

## Results

Our initial model is based on the observation that individuals living in areas of endemic *P. falciparum* develop immunity to malaria with age and exposure [25]–[27]; as levels of immunity are higher, there is a decline of parasite density in the blood [28] which is assumed to lead to milder clinical symptoms [25], [26], [29], [30], and to either lower probabilities of transmission to mosquitoes [31], [32] or alternatively, to higher transmissibility of asymptomatic carriers [33]. Thus, given that all infected individuals contribute (at least to some extent) to the general pool of the force of infection [34], but that for different immunity classes the selection pressure acting for or against drug resistance can differ, it is possible that under different transmission intensities drug resistance may or may not spread.

**Figure 1:**
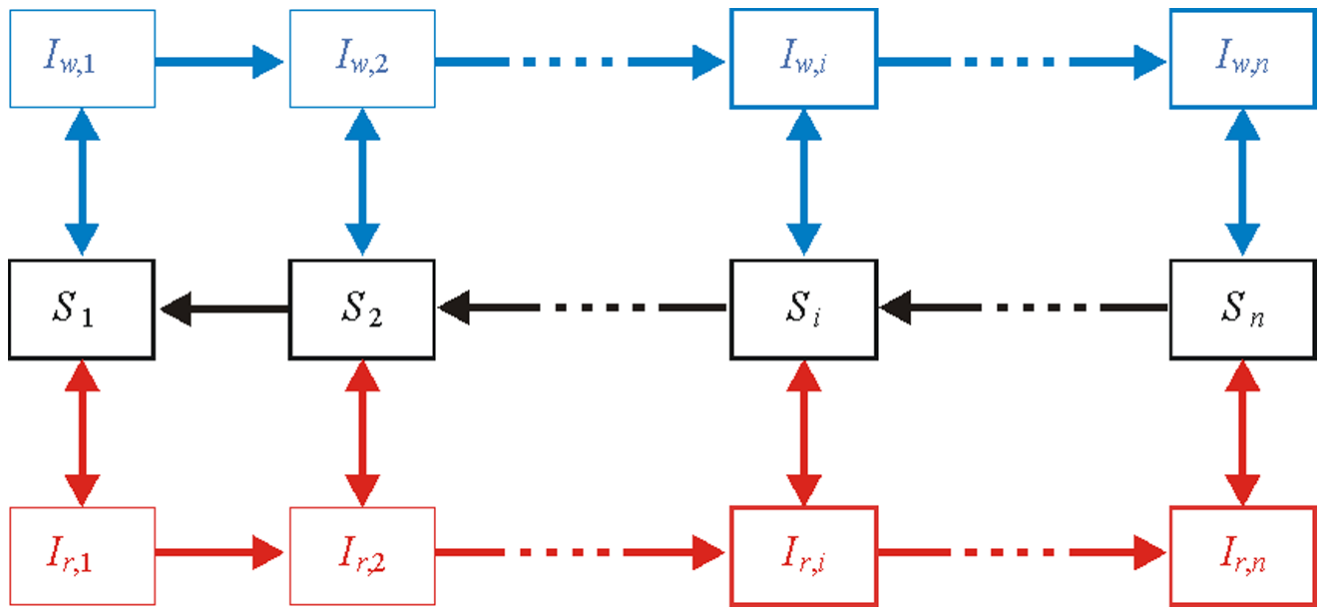
An illustration of the structure of the model. Immunity classes are portrayed in an escalating order from left to right, such that subscript *i* = 1 represents hosts with the lowest level of immunity, and subscript *i* = *n* represents hosts with the highest level of immunity. In each immunity class individuals are either susceptible or infected. If the duration of infection within a given class is relatively short, individuals recover and return to the susceptible state within their immunity class. When the duration of infection is extensive, or alternatively, if individuals are repeatedly re-infected spending large fractions of time in the infected state, it is assumed that this prolonged cumulative exposure leads to a higher level of immunity, moving these individuals to the next immunity class. However, if individuals in an immunity class remain susceptible for a long duration time, without renewed exposure to the disease during this time, they loose their current level of immunity and move back down the previous immunity class. The structure of the model captures the idea that acquired immunity gradually weakens in the absence of exposure to infection, but quickly strengthens itself if additional exposure accurse within a given time frame from the previous infection as discussed by [60]. Black represents susceptibles, blue sensitive wild-type and red resistant.

The model is constructed as a multi-class SIS model, with levels of immunity increasing from class to class (see diagram in Figure 1). The acquisition of immunity expresses itself in several ways. When immunity is low, infected individuals usually suffer from severe clinical symptoms, leading to a higher likeliness of drug treatment. However, as immunity is gained, these symptoms become milder and the use of drug treatment declines. It is assumed that as a consequence of the lower levels of parasitaemia that accompanies higher levels of immunity, infectivity to mosquitoes decreases.

We consider that an infection can take one of two forms; the infection is either by a drug sensitive parasite, the wild type, or by a drug resistant parasite. We assume a fitness cost accompanies resistance [35]–[37], which we assume to occur in the clearance rate of an infection, such that resistant parasites are naturally cleared more effectively from the host than the wild type. Note however, that the fitness cost accompanying resistance may occur in one or in several other stages of the parasites' life cycle, as discussed later on. Our modeling approach is an extension to that offered by Klein *et al.* (2008), with the added realism of multiple classes to approximate a continuum in levels of parasitemia and immunity. A detailed description of the model and the related equations is given in the Materials and Methods section.

**Figure 2:**
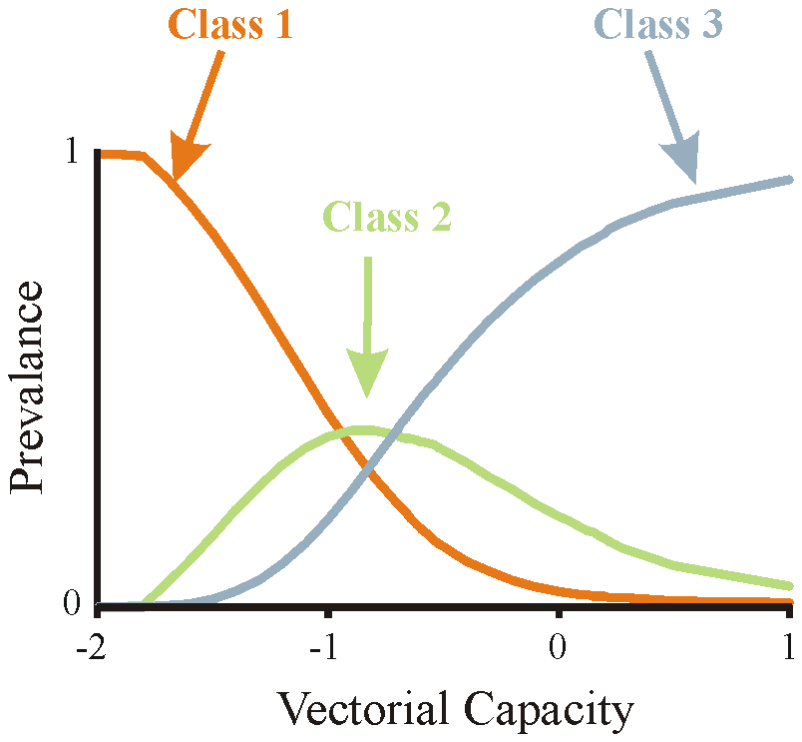
Fraction of the population in the different immunity classes as a function of vectorial capacity for a simplified three class model (the x-axis is given in log scale). The orange line represents the fraction of hosts in class 1, the green line represents the fraction in class 2 and the blue line represents the fraction in class 3. For low levels of vectorial capacity the majority of the population is in the lowest immunity class, and as vectorial capacity increases, individuals in the population gain higher levels of immunity.

Model simulations show that at high levels of transmission intensity, or vectorial capacity, the general prevalence of infection in the population is higher, and the distribution of individuals tends to be accumulated in the higher immunity classes (see Figure 2). As vectorial capacity increases, individuals are repeatedly bitten, which leads to higher rates of re-infection. The individuals that are rapidly re-infected will stay longer in an infected state. This leads individuals to gain higher levels of immunity, as well as insuring that the immunity they have gained is less likely to be lost. In return, larger fractions of the population exhibit milder clinical symptoms, and the relative drug use is generally lower. It is important to note that as the distribution of hosts leans towards the higher immunity classes, it is only the relative fraction of treated individuals which starts dropping, while the overall drug treatment in the population may continue to increase with the general rise in prevalence. This is in contrast to earlier notions that the decrease in drug pressure in high transmission areas is due to a general decrease in drug use and not a relative one [12], [38].

As expected, our results show that the resistant parasite has a clear advantage over the wild type when high levels of drug treatment are being applied. In this regime, if the drug pressure is high enough in relation to the fitness cost of resistance, the wild type is cleared from the infected hosts at a faster rate than the resistant parasite. Thus, the resistant parasite has the opportunity to spread more effectively throughout the host population, leading to the extinction of the wild type. However, as demonstrated in Figure 2, when vectorial capacity increases even further, larger fractions of the population accumulate in higher immunity classes for which clinical symptoms are milder. This leads to a drop in the relative level of drug treatment per infection, such that the drug pressure on the parasite decreases down to a threshold where the fitness cost of resistance is more dominant than the benefit of being resistant. At this point, the duration of infection by the wild type is effectively longer than that of the resistant, leading to the extinction of the resistant parasite. These results are similar to those obtained by Klein *et al.* 2008 based on their two-class SIS modeling approach. We ask next under what conditions, if any, would the wild type refuge provided by clinical immunity be lost for higher levels of transmission, restoring the advantage of resistance.

**Figure 3:**
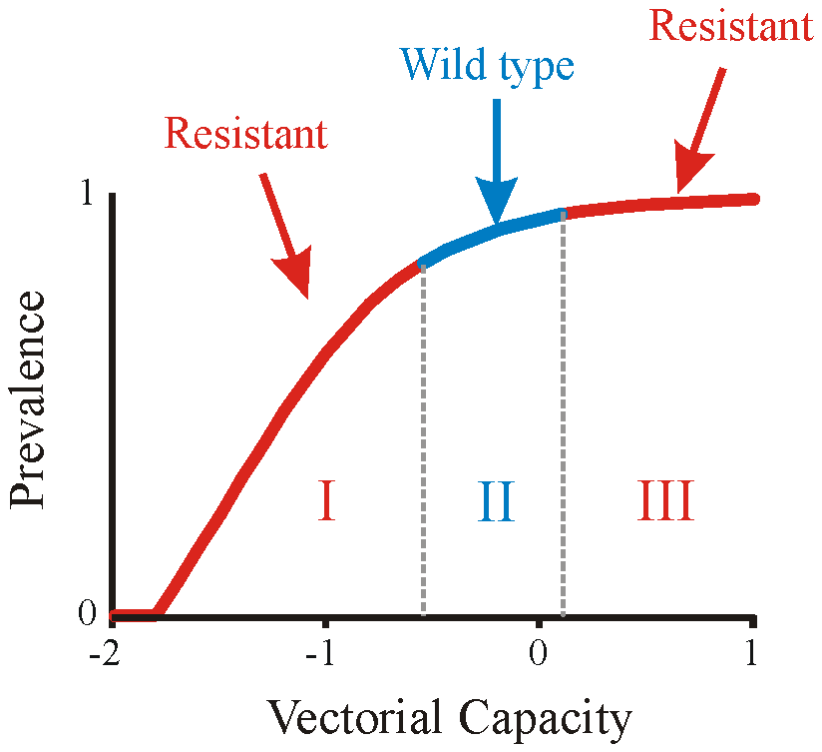
Non-monotonic pattern of resistance spread with transmission intensity. The fraction of infection in the population as a function of vectorial capacity for a simplified three-class model (the x-axis is given in log scale). Red indicates infection by the resistant parasite; blue, by the wild-type parasite. For very low levels of vectorial capacity, both parasites do not persist (the effective reproductive ratio, *R*_w_ and *R_r_*, of the wild-type and resistant, respectively, is smaller than 1). With the increase of vectorial capacity, the *R_r_* of the resistant parasite is larger than that of the wild type, *R*_w_, making it dominate the infected population (region I, marked in red). At intermediate vectorial capacities, a first threshold is observed where the wild type parasite out-competes the resistant one (region II, marked in blue). At high levels of vectorial capacity, a second threshold is observed where the resistant parasite out-competes the wild type (region III, marked in red). Parameters are given in Table 1.

We find that one way to obtain this pattern in the multi-class SIS model, is to consider that the duration of infection is longer as immunity increases. Lower levels of parasitemia in the blood, as well as the mild clinical symptoms, may decrease the severity of the immune response, thus allowing infections to last longer. Evidence for this possibly counter-intuitive assumption is discussed in the Discussion section and implications of releasing this assumption are considered later in this section. When it applies, a second threshold can exist, where an additional turnover takes place (see Figure 3). Specifically, when the duration of infection is sufficiently longer in the higher immunity classes, the fitness cost of the resistant parasite becomes negligible in comparison to the drug pressure that is still being applied on the population, even if at very low levels. In Figure 3 we demonstrate by simulation a case in which there are two such turnovers; from a regime in which the resistant parasite dominates the population due to strong drug pressure, to a regime in which the wild type takes over due to the fitness cost for the resistant parasite, and back to a regime in which the resistant parasite dominates the population again, due to a natural increase in the duration of infection. Formal analysis of this result is given in the Materials and Methods section, where the effective reproductive number (*R*_0_) of the wild type and resistant parasites is calculated.

The non-monotonic pattern of resistance spread with transmission intensity in Figure 3 can be modified by varying the values of three key parameters in the system: (***i***) the levels of treatment in the population, (***ii***) the fitness cost on the resistant parasite and (***iii***), the natural duration of infection (see Materials and Methods, necessary conditions in a 3-class model, as well as Figure S1-1 and S1-2 in Supporting Information S1 for 2D projections of varying costs of resistance and treatment levels as a function of vectorial capacity). In other words, the existence of the above-described thresholds depends on a sufficiently high (intermediate) cost of resistance and on a sufficient level of drug treatment. The fitness cost of resistance is likely to vary for different anti-malarial drugs [35].

In the context of our model so far, a non-monotonic pattern with drug resistance parasite favored at low and high transmission intensities, and the sensitive parasite at intermediate values, can only arise under the assumption that the duration of infection increases with immunity level. (We note, however, that the first threshold does not require this assumption, and occurs also under a constant [1] or decreasing duration.

We specifically note that both our model and that of Klein and colleagues, implicitly consider that the rate at which immunity is boosted and lost by re-exposure is constant for all levels of transmission intensity. This ignores the role of strain (antigenic) diversity in the acquisition of immunity, which differs between regions, and under different transmission intensities. In endemic areas, where vectorial capacity is high, strain diversity is also expected to be higher [39], [40], and so the acquisition and maintenance of immunity in these areas is lower than it would be in areas of low diversity. We therefore consider that 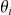, i.e., the rate at which immunity is gained, is a function of parasite diversity. Thus, the rate of moving from one class of immunity to the next depends on the level of parasite diversity, and therefore, in the model itself on transmission intensities.

**Figure 4:**
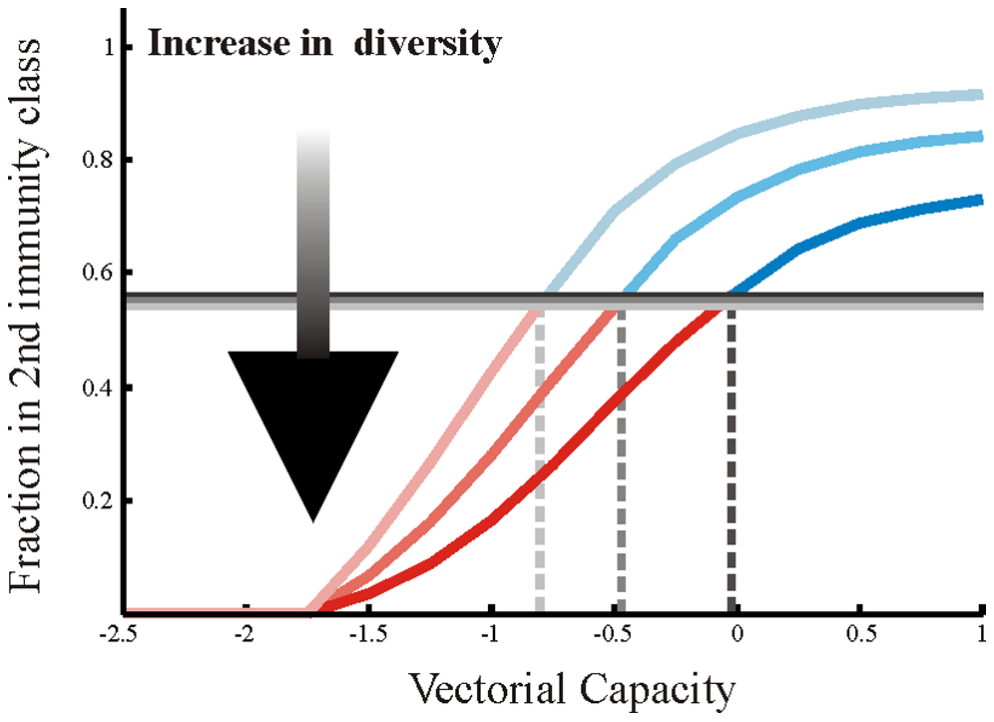
Fraction of clinically immune individuals as a function of vectorial capacity for a two-class model scenario (the x-axis is given in log scale). Results of increasing strain diversity are plotted in escalating shades from top to bottom (gain of immunity 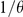 = 5 years, 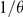 = 10 years and 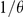 = 20 years, respectively). Threshold levels at which resistance is out competed by wild type, 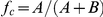, are marked by horizontal gray lines (see definition of *A* and *B* in the Materials and Methods subsection, Thresholds in a two class model). For lower strain diversity, immunity is rapidly gained in the population and the threshold, 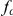, is low. Alternatively, when strain diversity is higher, immunity is gained more gradually, and the threshold is higher (Similar results are obtained for multiple classes).

Figure 4 illustrates the role that strain diversity may have on the establishment of anti-malarial drug resistance. The expected threshold (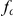) for a two-class model is 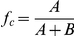, where *A* and *B* are positive and as defined in the Materials and Methods section (Thresholds in a two class model). Hence, when the fraction of the population in the second immunity class (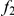) is below this threshold (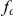), the resistant parasite out-competes the sensitive one, and when the fraction is above this threshold, the sensitive parasite out-competes the resistant. For different diversity levels, we find that the lower the diversity, the lower the vectorial capacity at which the threshold is crossed. Hence, the first threshold described in our analysis (as well as in [1]) can become *a moving target*, shifting towards higher values of transmission intensity as diversity also increases. This would make such threshold more difficult to reach with the population remaining in a regime where drug resistance is favored as transmission intensity increases.

**Figure 5:**
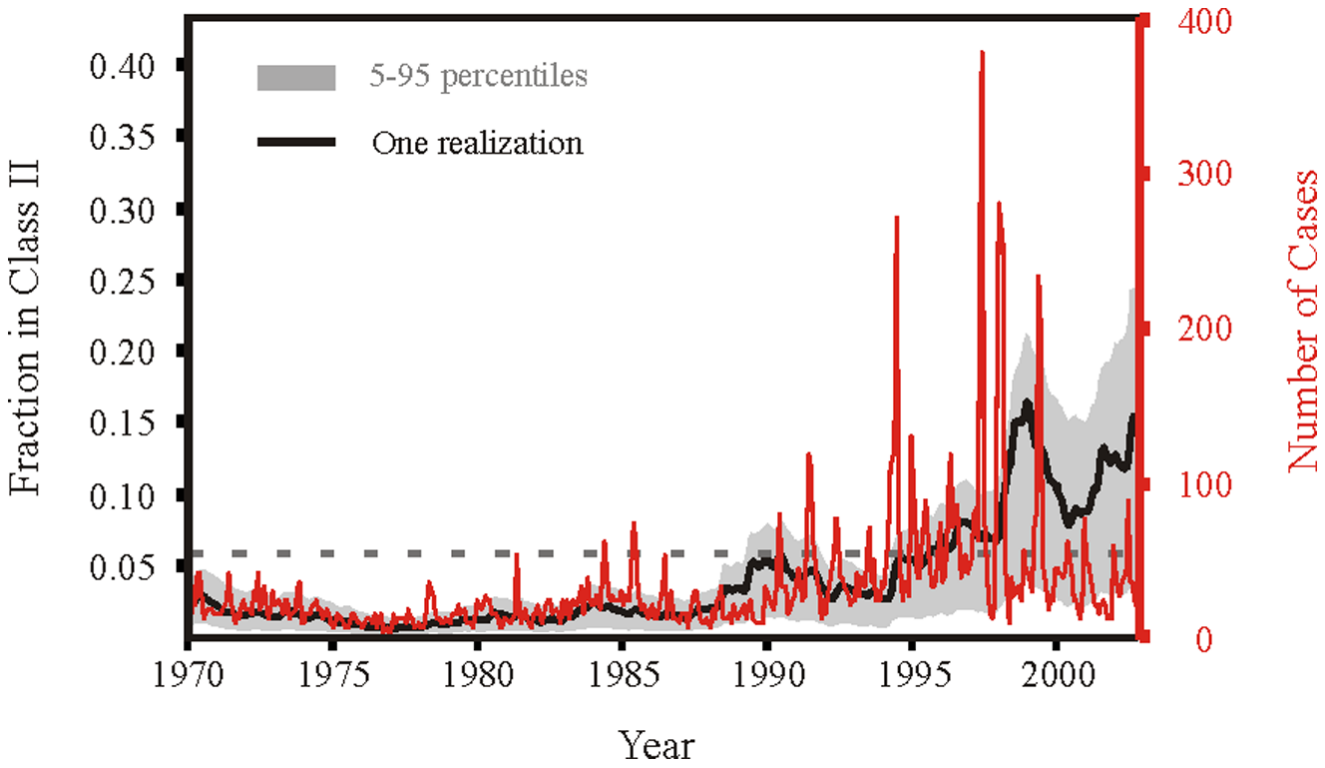
Simulated trajectories of the two-class model with the force of infection driven by a local temperature and rainfall records through a sub-model for the dynamics of infection in the mosquito vector. Parameters of the coupled mosquito-human equations were obtained by fitting this model to the monthly time series of confirmed cases from the hospital serving the tea estates of the Brooke Bond Farms in Kenya (elevation 1780–2225m) from 1970 to 1985 only, a period of low incidence when drug resistance was not apparent in this region. The search of parameter space produces sets of values, reflecting the uncertainty in their determination given the data (see Supporting Information S2). Simulations of the model for the different parameter sets and with the observed temperature, produces the range of behaviors illustrated in grey for the fraction of the population in the clinically-immune class (5% and 95% percentiles of the distribution for the predicted fraction of hosts in the second immunity class at each time. The horizontal dashed line represents the threshold fraction of hosts in the second immunity class (*fc*) below which drug resistance is favored, and demonstrated in black is a realization with fitness cost of 0.5, as explained in Materials and Methods, Thresholds in a two class model. In red is the time series of the number of observed cases.

Because Kericho is in an area of low transmission intensity, at the edge of the altitudinal distribution of the disease, we can consider the simpler version of the model, with only two levels of immunity, focusing on the first threshold. This reduces the number of parameters and allows us to extend the model to consider the vector's dependence on temperature. We note that in this version of the model we do not specify a priori how the duration of infection varies with level of immunity. The force of infection is specified by coupling the model for the human host to a vector sub-model describing the abundance of mosquitoes in four classes, corresponding respectively to larvae, uninfected, exposed and infected adults. The full set of equations for the coupled human-mosquito model are given in Supporting Information S2. The parameters of this coupled model are fitted to the malaria time series from 1970 to 1985 only, using as covariates local meteorological time series for rainfall and temperature [41]; see also Supporting Information S2). Importantly, consideration of this time period, allows us to fit the model with only the wild-type strain. The fitted parameters and the observed temperature data are then used to simulate the model forward in time to quantify the consequences of the observed trend in temperatures of approximately one degree (see Figure 1 in [9]). We specifically address whether the resistant strain would have a higher fitness and therefore be able to invade the system as temperature increases. Details on the method of parameter estimation, based on maximum likelihood estimation via a genetic algorithm, can be found in Alonso *et al.* (*in review*) and in Supporting Information S2. It is important to note that given the complexity of the model, our search of parameter space gives us not one but a family of parameter sets with equivalent likelihoods. This set reflects the uncertainty in the estimated parameters, especially given our reliance on the first 15 years only, which exhibited weak seasonal transmission and therefore provide limited information. We generate based on this set, a family of simulations whose behavior is represented in Figure 5a. Despite the uncertainty, this figure shows a clear tendency of the population in the clinically immune class to increase from the 1970s to the 1990s, concurrent with an increase in the size of epidemics. Provided the first threshold has not yet been crossed, these changes would imply initially an increase of the effective *R*_0_ of the resistant strain, at a faster rate than that of the wild-type, and therefore, an increasing ability of the resistant strain to invade and displace the resident wild-type (see Figure S3-1 in Supporting Information S3) leading to a synergistic effect of climate change and drug resistance increasing the number of symptomatic cases of malaria. We note, however, that the difference between the respective *R*_0_ of the two strains is not monotonic below this threshold, and that after the initial increase (synergy), this difference reaches a maximum and then decreases as vectorial capacity rises further (see Figure S3-1 in Supporting Information S3).

Whether or not the system has crossed the threshold depends on two unknown quantities, namely the cost of resistance and the proportion of the recovery rate that is due to treatment (see Supporting Information S2). We can consider different combinations of these two quantities and obtain the fraction of simulated solutions that have not crossed the threshold at a given point in time. This fraction quantifies the likelihood that the resistant parasite can invade the system and spread, given the uncertainty in the fitted parameters. Figure S2-1 (Supporting Information S2) shows a general tendency for this fraction to decrease in the 1990s. However, for high treatment levels and low to moderate costs of resistance, this probability remains above 0.5, implying that clinical immunity has not increased enough to tip the balance in favor of the wild-type by creating a refuge, an that the majority of the solutions have not crossed the threshold. It is also possible for the solutions to cross the threshold back and fourth intermittently as vectorial capacity varies seasonally and interannually. This occurs at intermediate values of the cost of resistance and, reflects a regime in which vectorial capacity is not high enough yet to provide a sustained refuge for the wild type.

That the system is not likely to have crossed the threshold that favors the wild type, tells us about invasion of the resistant strain (technically that we are in a region where its reproductive number is higher than that of the wild-type), but does not tell us whether this advantage has become larger with the increase in transmission intensity. This is because, as we noted before, this difference is non-monotonic for values of transmission intensity below the threshold. In Figure S2-2 (Supporting Information S2) we consider this specific difference and illustrate the tendency for this difference to increase from the 1970s to the 1990s provided treatment levels were sufficiently high.

## Discussion

Our findings underscore that drug resistance and climate change can interact through the effects of warmer temperatures on transmission intensity. The non-monotonic pattern described here as the result of two thresholds in the selective advantage of resistance (see Figure 3, and pattern ***a*** in Figure S1-1 in Supporting Information S1), resembles the ‘valley phenomenon’ observed for CQ resistance in Uganda, where treatment failure was found to be higher in areas of low and high transmission, and at its lowest in areas of intermediate transmission [22]–[24]. The epidemiological model can also explain other patterns such as a monotonic increase in drug resistance as a function of transmission intensity (see pattern ***e*** in Figure S1-1 in Supporting Information S1) resembling the observations found for sulphadoxine-pyrimethamine (SP) resistance in the same region [22]–[24]. Key parameters determining the existence of the two different thresholds as transmission intensity increases are the cost of resistance for the different drugs and the levels of drug treatment. We have also shown that increases in parasite diversity with transmission intensity can increase the value of the first threshold, and therefore, delay or eliminate the emergence of a refuge for the wild type at high transmission.

The question of whether drug resistance is more likely to spread in areas of low transmission intensity potentially carries major implications on control measures such as the use of bed nets and insecticides. Only under a positive association, vector control strategies would work hand in hand leading both to a decline in the general prevalence as well as limiting the spread of drug resistance keeping drug treatment effective. Klein *et al.* (2008) discuss the consequences for malaria control, especially in high transmission (endemic) regions, as the result of the emergence of a wild-type refuge at high transmission intensities. However, further examination of their two-stage immunity model and consideration of multiple levels of immunity shows here that the existence of a refuge (and its associated threshold) depends on a number of important considerations that require quantification for specific settings. In particular, it is possible for a relatively small reduction in transmission intensity to favor the wild type, but for an even larger reduction in transmission intensity to give rise to the opposite effect. Anti-malarial drug resistance could be inhibited in the early stages of control programs, only starting to resurge as the disease nears eradication.

From the perspective of climate change, the interplay of transmission intensity with the evolution of drug resistance is potentially most important for epidemic malaria in highland areas at the edge of the distribution of the disease, where temperature has limited transmission in the past. Regions previously considered epidemic in the highlands of Kenya have shown a tendency towards more seasonal outbreaks, with signatures of more endemic behavior in the age distribution of cases [6], [42]. A decrease in the adult-to-child ratio of inpatients in the mid-1980s reflects an increase in the levels of immunity in the population [6], [42]. These observations are consistent with our results for the Kericho tea plantation showing an increase in the fraction of the population with clinical immunity from the 1970s to the 1990s. We note that this increase is in our projections for the 1990s purely the result of the observed increase in temperatures. The implication is that warming was sufficient to produce a significant shift in the epidemiological structure of the population. Other factors must also be at play, however, since the concomitant increase in cases produced by our model simulations is significant when compared to the no trend scenario, but is typically smaller than that of the observed cases [41]. To the left of the first threshold, the increase in transmission intensity from the low values in the 1970s is likely to have been accompanied, at least initially, by an increase in the effective reproductive number of the resistant phenotype at a faster rate than that of the wild-type strain. It is in this sense, that to the left of this threshold, warmer temperatures can act synergistically with drug resistance.

This raises the question of whether the temperature trend was sufficient to push the system across the first threshold identified in the two-class model for which the resistant phenotype is no longer favored and a wild-type refuge is established. This is not supported by the reported 50% treatment failure for children in the hospital in the 1990s, which indicates the presence of drug resistance in this region [7]. The location of the system relative to this threshold depends in the epidemiological model on the levels of treatment and costs of resistance for chloroquine. Treatment levels are likely to be high in this tea plantation where the hospital has provided health care for the population of workers and its dependents. Under this scenario, our results suggest a high likelihood that the threshold has not been crossed. Very high costs of drug resistance would have been required for the wild-type refuge to emerge under high treatment levels.

Our analysis considered the long-term outcome of the competition of the two parasites, addressing whether or not the resistant parasite would be able to invade if transmission intensity was kept constant at its value for each month. Thus, seasonality and non-stationary temperature conditions are considered for the population dynamics of the resident strain, with the effective *R*_0_ of this strain evaluated at the equilibrium of the model for those conditions at each time, but not for the transients of competition between the two strains. It is an open question whether such transient dynamics are involved in the seasonal variability in the frequency of the resistant genotype observed for other epidemic regions in desert regions of Africa [43], [44]. We are not aware of similar observations for highland regions but sampling resistance levels, and not just cases, in surveillance efforts over time would allow a better parameterization of epidemiological dynamics and a better understanding of drug resistance in the context of variable transmission.

Epidemiological parameters were obtained here by fitting the model, with only two classes, to the first part of the malaria time series when incidence was low and drug resistance was not apparent in this region. It is important to note that the model was not fitted to the whole time series, and in particular to the epidemics of the 1990s during the period of higher incidence, because our aim was not to consider *a priori* that the observed temperature trend was the only factor responsible for the observed exacerbation of the disease. It would have been trivial to fit the model to the whole time series; however, this would have also been completely uninformative in a place where many other factors, other than temperature, are known to have varied concurrently with temperature. We used the model to ask instead what would have been the possible effect of the observed temperature trend in the 1990s, given values of the transmission intensity and other parameters consistent with the observed data in the 1970s and beginning of the 1980s.

It is also important to mention that our results for Kericho do not imply that the introduction of a new drug would be ineffective. In fact, the introduction after the 1990s of new drug treatments, other than chloroquine, has been proposed as one main reason for the decrease in malaria cases in more recent years [7]. Other control measures including insecticide spraying have also been implemented in the region.

The epidemiological models considered here complement theoretical studies based on population genetics, and the clear importance of epidemiological considerations related to immunity here and in other recent studies [1], [14] supports the recent call for models that merge population dynamics and population genetics [45]. Field evidence relating the transmission intensity to the spread of drug resistance remains limited, with observations leading to contradicting interpretations [22]–[24], [46], [47]. This is also the case on the theoretical side, with previous mathematical models based on population genetics suggesting a positive association [10], [48], a negative association [49]–[51] or a concave association [13]. Critical assumptions in these models relate to the number of genes encoding resistance [10], [11], [52], [53], the existence of intra host dynamics [12] and a direct tie between transmission intensity and recombination rate via the level of clonal multiplicity [13]. Further empirical work is needed to test these assumptions [13], especially at the interface of within-host and between-host dynamics.

Only the existence of a second threshold, for which the advantage of resistance is restored at high transmission intensity, is dependent in our theoretical results on the assumption that the duration of infection increases as individuals acquire higher levels of immunity. Although most epidemiological models assume the opposite relationship, there is little empirical evidence on the effect of immunity on the duration of infection, despite the central relevance of this parameter to vectorial capacity and the basic reproductive number of the disease, as pointed out by [54]. There is also considerable uncertainty in the related pattern between the duration of infection and age (see for example, Table S2 row IM10 in supplement of [14]). Because earlier studies demonstrating shorter infected periods with age were based on analyses of sequential blood slide data [55], [56], these were limited by the problem of discriminating between successive new infections and persistent ones [57], [58]. A more recent statistical analysis of infections characterized by molecular methods at the level of the parasite's genotype supports the opposite pattern of an increase in the duration of infection with age [58]. Indirect evidence for this pattern was also found in comparisons of age-prevalence curves between dry and rainy seasons, and in a malaria transmission model that explores the biological basis for acquired immunity and differentiates ‘anti-disease’ and ‘anti-infection’ protection [57]. Ultimately, the relationship between immunity and epidemiological parameters will be elucidated by a better understanding of antigenic strain structure, within-host antigenic variation, and strategies for immune evasion of the parasite.

We have assumed that the fitness cost accompanying resistance occurs in the clearance rate of an infection, such that resistant parasites are naturally cleared more effectively from the host than the wild type. It is however possible, for the fitness cost of resistance to occur in one or in several other stages of the parasites' life cycle, resulting for example in impaired within-host growth, or lower transmission probabilities. It is straightforward to extend our analyses to consider the robustness of the valley phenomenon to these variations (see Supporting Information S4). Interestingly, an increase or decrease in the transmission probabilities (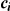
's) between the immunity classes plays no role on the conditions for the valley phenomenon. Moreover, when there is no fitness cost on the probability of transmission, the clearance rate of the resistant parasite must be assumed to decrease with gain of immunity. This is the case, however, regardless of whether the cost of resistance decreases or increases clearance rate. Finally, if the fitness cost acts on transmission probabilities, then the valley phenomenon may emerge even when there is no reduction in clearance rate between the immunity classes (of both the resistant and the wild type).

These considerations emphasize the need for a better understanding of the mechanisms operating in the within-host dynamics of the pathogen that underlie specific epidemiological assumptions. For example, a fitness cost that impairs within-host growth rates may also lengthen the duration of resistant infections, or change the transmission probabilities. A different epidemiological consideration concerns the duration of infection with level of immunity. The relation between these two effects on clearance rates is not straightforward, and ultimately only an empirical understanding of within-host mechanisms can elucidate this, including the possibility of inconsistent hypotheses. We have considered here a particular model structure motivated by the previous work by Klein *et al.* (2008), to represent the acquisition of clinical immunity and different, associated levels of parasitemia. Future work should examine the robustness of our results to model structure, as the representation of these complex biological phenomena in a disease such as malaria remains a challenge.

As shown by the detailed age-structure model of Pongtavornpinyo *et al.* (2008), empirical data on the duration of infection and other functions relating immunity to central epidemiological parameters are critical to models that consider the spread of resistance, especially for new drugs and multi-drug treatments (such as ACT). Empirical studies that consider asymptomatic individuals and molecular methods of parasite detection would be most useful for these purposes. Additional field data on temporal and spatial patterns of drug resistance would be clearly invaluable; their interpretation is likely to require consideration of an epidemiological perspective at the population level.

## Materials and Methods

### Human dynamics

We assume that there are three possible states in each immunity class; susceptible, infected with a sensitive ‘wild type’ parasite, and infected with a resistant parasite. The human population densities of the different states of each class are denoted as 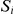, 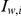, 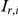, where the subscript *i* = (1,…,*n*) denotes the immunity class. The subscripts *w* is used to denote an infection by a ‘wild type’ parasite, while the subscripts *r* is used to denote an infection by a resistant parasite. The natural birth and death rate are assumed to be equal, such that the population birthrate *B* is equal to the per capita death rate of the human population, 
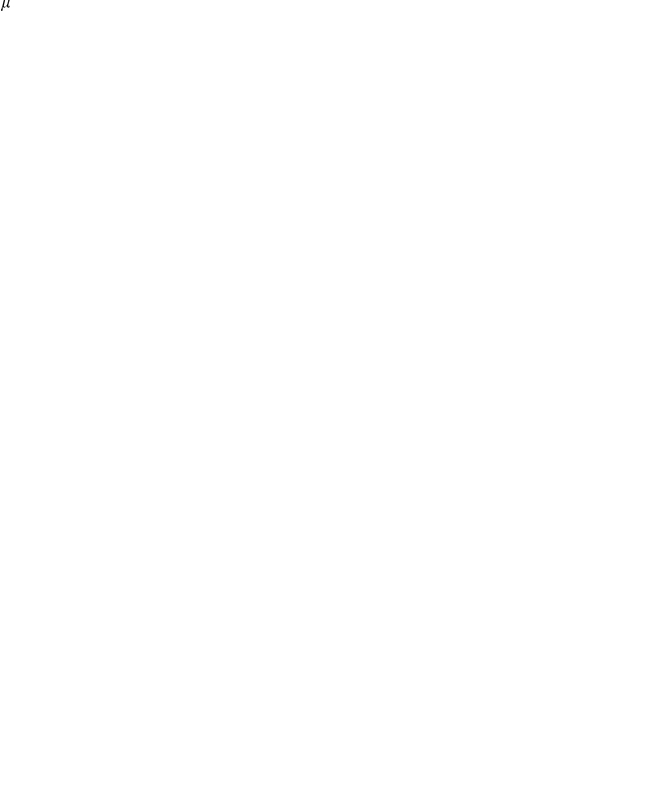
. Disease induced mortality is allowed to vary between immunity classes and we assume that 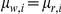 or all immunity classes.

### Vectorial capacity

As in Klein *et al.* (2008), model notation follows [3], [59]; *m* denotes the number of mosquitoes per human and *a* the human feeding rate (the number of bites on humans per mosquito per day). The instantaneous death rate is *g*, and 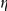 is the number of days required for sporogony. Vectorial capacity, *V*, i.e., the number of infectious bites by a mosquito over its lifetime, is then given by the formula 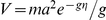. The fraction *P* of bites on humans that infect a mosquito depends on the transmission probabilities of the different immunity classes, *c_i_*, and the fraction of infected hosts in each immunity class, such that 
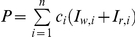
. The sporozoite rate, i.e., the fraction of infectious mosquitoes, is defined as 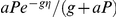. The entomological inoculation rate, EIR, i.e., the number of infectious bites per person per day, is calculated as the product of the human biting rate, *ma*, and the sporozoite rate (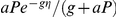). The force of infection, or happenings rate, *h*, is defined as *b*EIR, where *b*, the infectivity rate, measures the fraction of bites in humans that produce a patent infection. It follows that 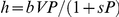, where 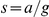 is the stability index, i.e., the number of bites on a human per vector per lifetime. The fraction of infections that are drug sensitive is given by 
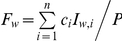
 and the fraction that is drug resistant is 
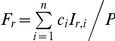
. Happenings rates for drug-sensitive and drug-resistant infections are 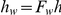 and 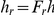, respectively.

### Acquisition of immunity and parasite clearance

Immunity level (*i*+1) is assumed to be gained for infected individuals in immunity level (*i*) after 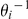 days. Once individuals gain immunity at level (*i*), protection is sustained through re-infection and is otherwise lost at a rate of 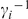 days. It is assumed that the rate at which infections are cleared by drugs is 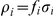, where 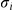 is the rate at which clinical symptoms arise and 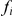 is the fraction of infections treated and cleared of parasites. Note that as immunity is gained, the likeliness that infections lead to clinical symptoms decreases, such that 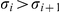. We assumed that the natural clearance rate of an infection by a resistant parasite (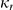) is faster than that of the wild type (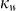), i.e., 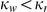. Thus, in the absence of any drug treatment, the wild-type will always out compete resistance.

### Equations

The model is defined by the following set of coupled ordinary differential equations:
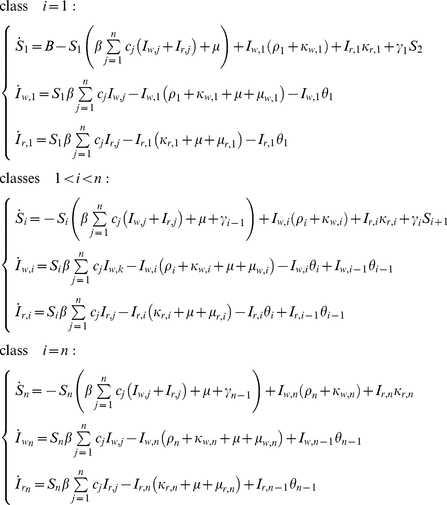
(1)where 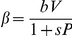.

### Calculating *R*_0_

Parasite fitness is calculated as the basic reproductive number (*R*_0_), both for the sensitive ‘wild type’ and for the resistant parasites. For each immunity class, *i*, the population was assumed to be completely susceptible within that class, such that 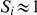. The *R*_0_ -values of the sensitive wild type parasites in each immunity class are:
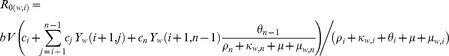
for 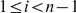, and:
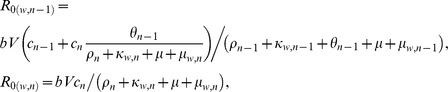
where 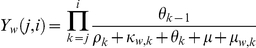.

Similarly, *R*_0_ -values for the resistant parasite:
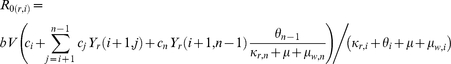
for 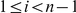, and:
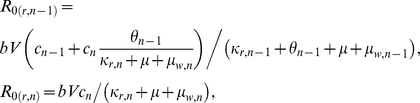
where 
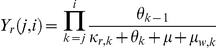
.

It is now possible to determine which of the two parasites will out-compete the other, by defining the effective reproductive rate of the sensitive and the resistant parasite, with respect:
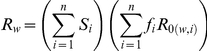
and
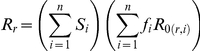
where 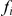 (>0) is the fraction of the population that is in immunity class *i* (i.e., 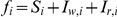). When 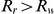 and 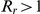 the resistant parasite spreads throughout the population and the sensitive parasite goes extinct. Thus, assuming that 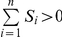, we find the following condition:
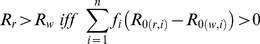
(2)


Clearly, when all immunity classes fulfill 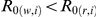, condition (2) is held, and if in addition if 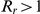, then the resistant parasite spreads throughout the population. We note that a necessary condition for the persistence of either the sensitive or the resistant parasite is that 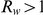 or 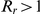, with respect, however, as demonstrated, this is not a sufficient condition.

Taking a closer look at condition (2), we see that it can still be met if only some of the classes fulfill 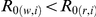, such that it is still possible that 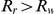, and this depends on two factors: **1)** the extent of the difference between the *R*_0_'s of each class, and **2)** the fraction of the population in each class (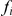).

The **first factor** is determined by the selection pressures acting on the two types of parasites. For example, when drug pressure is high, as would be expected for small *i*'s, then 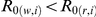. But when drug pressure drops in the higher immunity classes due to the reduction in clinical symptoms, the fitness cost of being resistant may start taking it's toll such that 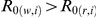. However, in the latest classes, when the duration of infection is extended, the fitness cost may loose it's impact, and again 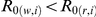. The difference between the basic reproductive numbers in each class keeps the same sign, either negative or positive, regardless of the level of vectorial capacity. When vectorial capacity is low, most of the population belongs to the classes of the lowest *i*'s (see orange curve in Figure 2), and thus, if 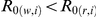 in these classes, it is likely that 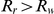. As vectorial capacity increases, a higher fractions of the population is now in the higher classes (see green curve in Figure 2), and if 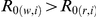 in those classes, it is possible that 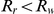. Similarly, if the vectorial capacity is even higher, most of the population will be situated in the highest immunity classes (see blue curve in Figure 2), and if 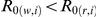 in these classes, it is possible to return to a case where 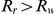. An example of such a case can be found in Figure 3.

### The 3-class model

#### Simulations

Simulations were carried out by using a Matlab ODE solver for the set of equations (1). Similar parameters to those used by Klein *et al.* (2008) were chosen, and defined in Table 1.

**Table 1:** 

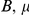	Natural human birth and death rate	1/(60*365)
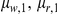	Disease induced death rate in immunity class 1 (0 for the other classes)	180/100000/365
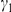	Loss of immunity rate	1/(3*365)
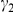		1/(2*365)
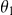	Gain of immunity rate	1/(5*365)
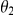		1/(10*365)
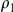	Rate at which existing infections are cleared by drugs ( 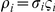 ) 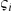 Fraction of clinical symptoms treated 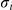 Clinical symptoms arise	1/200
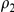		1/500
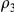		1/500
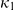	Natural clearance of infection rate	1/150
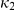		1/150
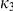		1/400
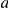	Human feeding rate	0.3
*B*	Infectivity rate	0.8
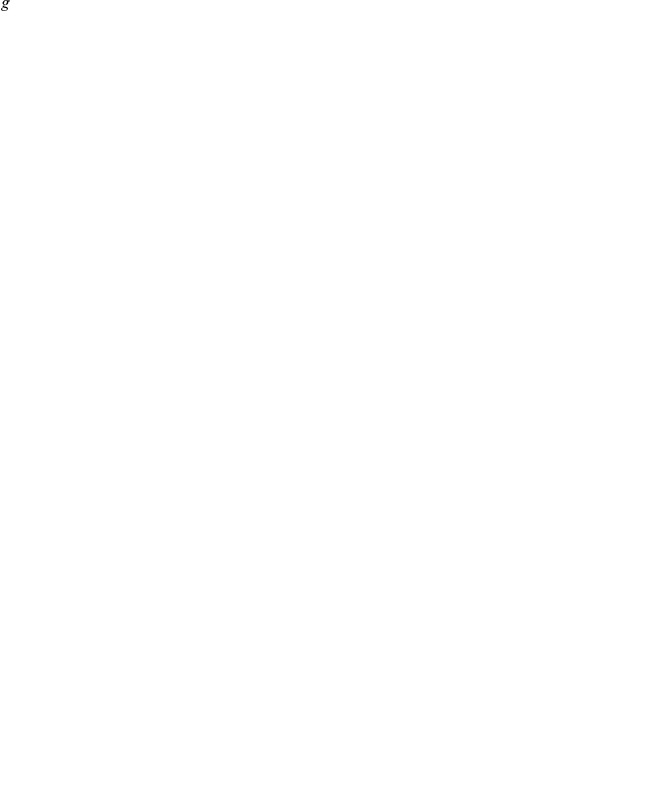	Instantaneous death rate	1/10
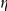	Number of days required for sporogony	10
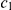	Transmission probability	0.7
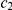		0.5
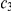		0.1
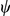	Fitness cost	0.6

Note that following Klein *et al.* (2008), we have assumed that transmission probabilities decrease with the gain of immunity. It is however possible that there is higher transmission probabilities in asymptomatic carriers (see for example, [33], and as we show further down our results are not sensitive to this assumption.

#### Necessary conditions for the non linear resistance pattern in the 3 class model

A necessary condition for the non linear resistance pattern is for selection to favor the resistant parasite over the wild type in the first and third immunity classes, while favoring the wild type over the resistant one in the second immunity class, such that according to (2): 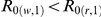, 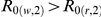 and 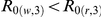. From the formulation of 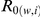 and 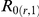 above, we find the following three conditions:
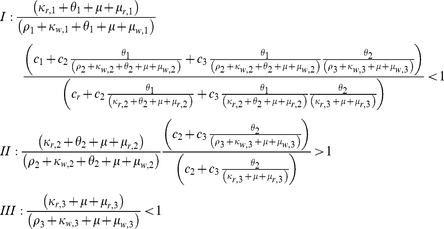
From condition III we find that 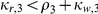, and thus, for condition II to hold we must have 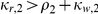. From this, in order for condition I to hold: 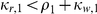. Equivalently, we find that the following three conditions for “valley phenomenon”: 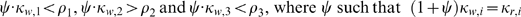


These conditions highlight three key parameters responsible for patterns of spread of drug resistance at different vectorial capacities, the fitness cost on the resistant parasite, the rate that infections are cleared by drugs and the natural clearance of an infection, 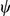, 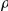 and 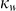, with respect. Note that these results demonstrate that the necessary conditions for the valley phenomenon do not depend on transmission probabilities decreasing or alternatively, increasing with the gain of clinical immunity, such that these results would hold both for 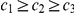 and for 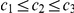.

### Two class Model

#### Simulations

As before, simulations were carried out using a Matlab ODE solver for the set of equations (1). Similar parameters to those used by Klein *et al.* (2008) were chosen, and defined in Table 2 (parameters not specified are identical to those in Table 1).

**Table 2:** 

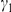	Loss of immunity rate	1/(3*365)
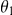	Gain of immunity rate	1/(10*365)
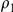	Rate at which existing infections are cleared by drugs ( 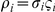 )	1/200
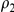		1/500
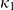	Natural clearance of infection rate	1/150
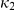		1/150
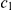	Transmission probability	0.7
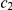		0.5

#### Thresholds in a two-class model

Similarly to Klein *et al.* (2008), for the two-class model, the effective *R*_0_'s of the sensitive and resistant parasites are:
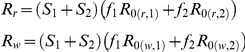
where
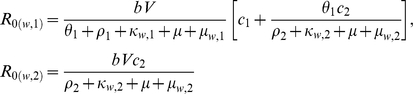
and
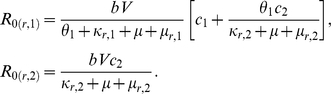
By defining:
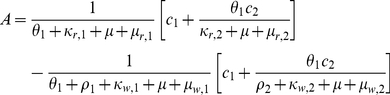
(3)and:
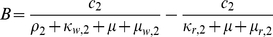
(4)we find that if *A* and *B* are positive, then 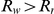 *iff* 
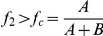
. Thus, when the fraction of the population in the second immunity class is below this threshold (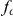), the resistant parasite out-competes the sensitive one, and when the fraction is above this threshold, the sensitive parasite out-competes the resistant. In Figure 5, the dashed horizontal line notes this threshold (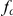) for the specific realization demonstrated in black. This threshold is calculated according to the parameters found for this realization with a fitness cost of 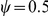 in the natural clearance rate of the resistant parasite.

**Figure 6:**
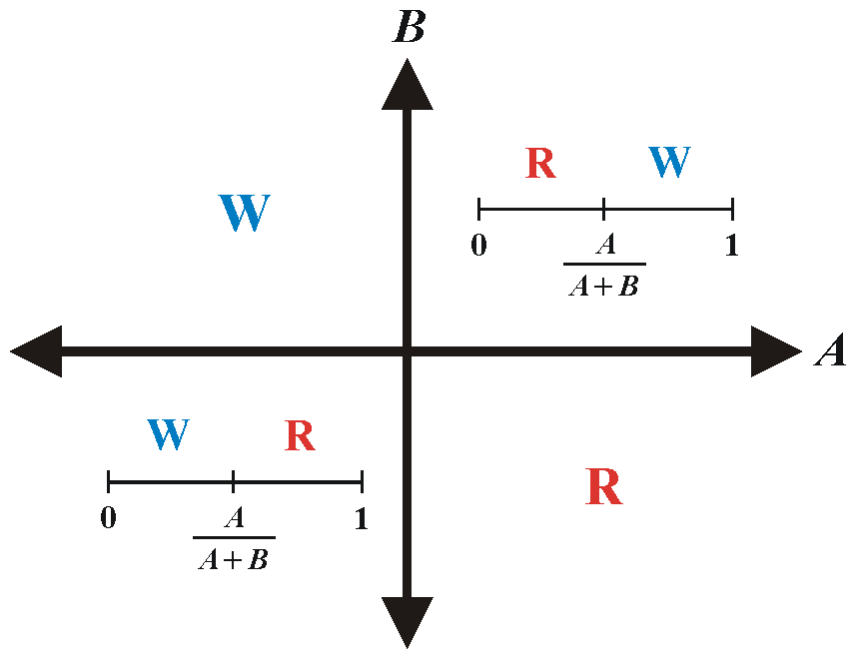
In the first quadrant, where *A* and *B* are both positive, when the fraction of clinically immune are below the threshold (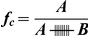), resistance dominates, and above the threshold, the wild type dominates. Reversely, in the third quadrant, when the fraction of clinically immune is low, wild type dominates, and past the threshold, resistance dominates. In the second and forth quadrants, there is no threshold, and the wild type and resistant parasites always dominate, respectively.

However, if *A* and *B* are not both positive we find that (see Figure 6):
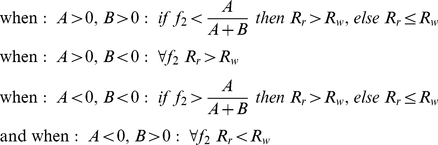



From these conditions, it is easy to find that if the fitness cost of the resistant parasite is smaller than 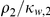, this is a necessary and sufficient condition for *B* to always negative (see quadrants 3 and 4 in Figure 6). However, the conditions for both *A* and *B* to be negative (quadrants 3 in Figure 6) are that the fitness cost is both smaller than 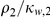 and sufficiently larger than 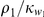.

## Supporting Information

Supporting Information S1 Key parameters.
(0.67 MB PDF)

Supporting Information S2 Linking the malaria model to temperature and rainfall data through a mosquito sub-model, and estimating the parameters using data of malaria cases and climate data.
(0.13 MB PDF)

Supporting Information S3 Relative effectively of the resistant parasite over the wild-type.
(1.37 MB PDF)

Supporting Information S4 A revised model.
(0.56 MB PDF)
