# Supplementary material for: Transmission Intensity and Drug Resistance in Malaria Population Dynamics: Implications for Climate Change
Source: PLoS One. 2010 Oct 26;5(10):e13588. doi: 10.1371/journal.pone.0013588 (PMC2965653; doi:10.1371/journal.pone.0013588)
Supplement: Supporting Information S1 — Key parameters. (0.67 MB PDF) [file pone.0013588.s001.pdf]

Transmission intensity and drug resistance in malaria population dynamics: implications for climate change

Yael Artzy-Randrup, David Alonso & Mercedes Pascual

<u>**Supporting Information 1**</u>

The non-monotonic pattern of resistance spread with transmission intensity in Figure S1-1 can be modified by varying the values of three key parameters in the system: (***i***) the levels of treatment in the population, (***ii***) the fitness cost on the resistant parasite and (***iii***), the natural duration of infection (see Materials and Methods, necessary conditions in a 3-class model). We consider first the fitness cost of resistance that is likely to vary for different anti-malarial drugs (Hastings and Donnelly, 2005) and establish a set of conditions for which the non-monotonic pattern emerges for a given drug. This pattern is denoted as pattern ***a*** in Figure S1-1. If a different drug is applied under the same set of conditions, whose fitness cost is higher, one of the following patterns emerges:

b) <u>All wild type (suppression of regions I and III)</u> - the resistant parasite never spreads, regardless of the level of vectorial capacity, and all infections are by wild-type parasites. Here, the prevalence of the wild type monotonically increases as vectorial capacity increases (Figure S1-1).

c) Resistant then wild-type (suppression of region III) - the resistant parasite dominates infections at low levels of vectorial capacity, and above a threshold, the wild type dominates all infections (Figure S1-1).

d) Wild-type then resistant (suppression of region I) - the wild type dominates infections at low levels of vectorial capacity, and above a threshold, the resistant parasite takes over.

Alternatively, we may consider that the cost of resistance to this second drug is lower, rather than higher. In this case, region II will be suppressed, leading to the following pattern:

e) All resistant (suppression of region II) - the wild-type never spreads regardless of the strength of vectorial capacity, and the prevalence of the resistant parasite in the population monotonically increases as vectorial capacity increases (Figure S1-1).

Note that similar results would be obtained if the level of treatment was varied (see Figure S1-2) being one of the key parameters outlined earlier.

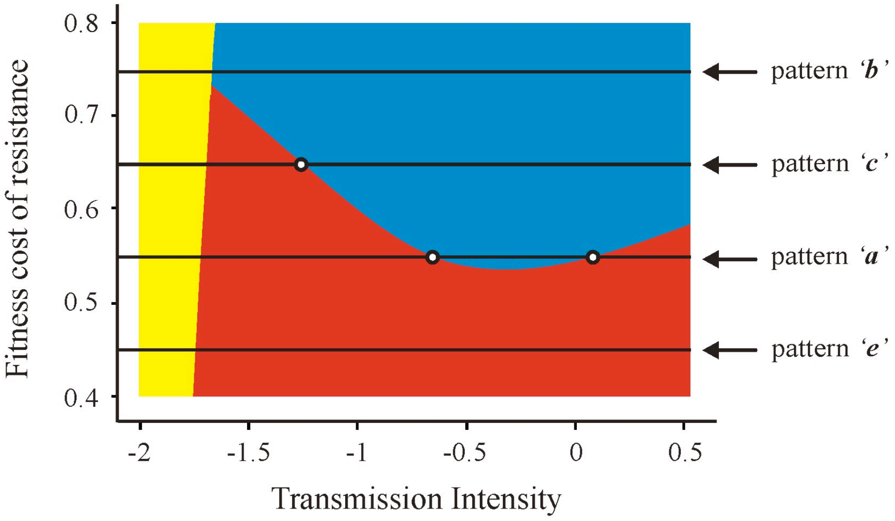

Figure S1-1**:** Bifurcation diagram as a function of vectorial capacity and fitness cost of resistance. The x-axis is given in log scale. Under different vectorial capacities (/levels of treatment, see Figure S1-2) there are three possible outcomes: spread of anti-malarial drug resistant parasites (red), spread of sensitive parasites (blue), or an infection free state (yellow). For different levels of the fitness cost the system exhibits different patterns in the shift between these outcomes as vectorial capacity increases. For low fitness cost e.g., 0.45, for all levels of vectorial capacity drug resistance spreads (see pattern ***e*** in text). For higher fitness cost, e.g., 0.55, the non-monotonic pattern emerges (see pattern ***a*** in text as well as example in Figure 3 in main text). Then, with a further increase in fitness cost, e.g., 0.65, drug resistance spreads at low levels of vectorial capacity and at a threshold level sensitive parasites take over (see pattern ***c*** in text) only the first threshold is observed. Finally, at high levels of the fitness cost, e.g., 0.75, drug resistance cannot spread, and for all levels of vectorial capacity all infections are drug sensitive (see pattern ***b*** in text). All other parameters are identical to those used for Figure 3, and are given in table 1.

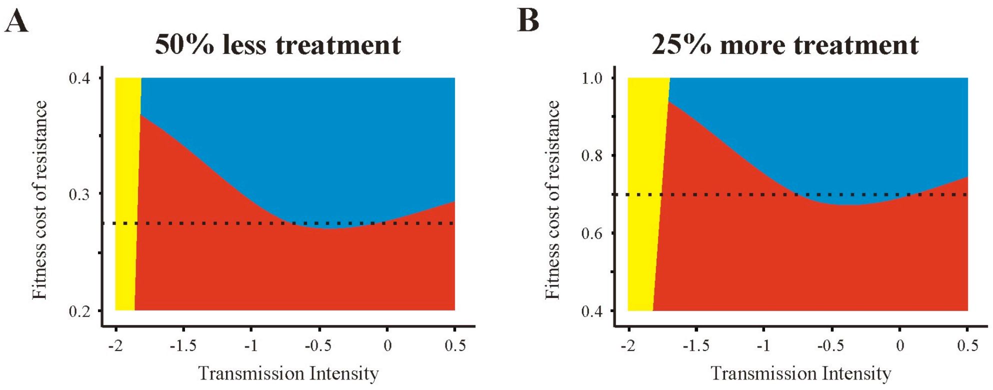

Figure S1-2**:** Bifurcation diagram as a function of vectorial capacity and fitness cost of resistance for two different levels of treatment. The x-axis is given in log scale. As demonstrated, the threshold level at which the wild type can invade changes under different treatment levels. Taking parameter values used in Figure S1-1, in A is the case where treatment is decreased by 50%, and in B, treatment is increased by 25%. When treatment is low, fitness cost of 0.275 on the resistant parasite is sufficient for observing the “valley phenomena”, while for higher levels of treatment, fitness cost on the resistant parasite would need to be at least 0.675 to allow the invasion of the wild type.
