# Supplementary material for: Transmission Intensity and Drug Resistance in Malaria Population Dynamics: Implications for Climate Change
Source: PLoS One. 2010 Oct 26;5(10):e13588. doi: 10.1371/journal.pone.0013588 (PMC2965653; doi:10.1371/journal.pone.0013588)
Supplement: Supporting Information S2 — Linking the malaria model to temperature and rainfall data through a mosquito sub-model, and estimating the parameters using data of malaria cases and climate data. (0.13 MB PDF) [file pone.0013588.s002.pdf]

# Drug resistance and climate change: Independent drivers of malaria dynamics? Supplementary Information

Yael Artzy-Randrup[1], David Alonso[2] & Mercedes Pascual[1*]
[1] University of Michigan, Department of Ecology and Evolutionary Biology
and Howard Hughes Medical Institute
[2] Community and Conservation Ecology Group
Center for Ecological and Evolutionary Studies
University of Groningen

# Supplementary Information

## Model Description

Our model describes a mosquito-human coupled system, where certain parameters are rain- and/or temperature-dependent. The temperature time series was obtained by dovetailing the records from two meteorological stations within the tea estate, together with adjustments for altitude based on mean temperature data from a number of stations in Kenya spanning a broader altitude range (see [1], for details). Two rainfall data sets were used in our analyses. The first one is a monthly rainfall time series from a local meteorological station in the Kericho district from 1970 to 2003 (Kericho Chagaik Estate, 0o20S, 35o20E, 6000ft; see Fig. 3 in [2]), and the second was from another meteorological station in the district from 1973 to 2003 (Hail Station, LAT/LONG/ 6480 ft; see Fig. S1 in [2]).

Malaria dynamics in humans extends the model in [3], and mosquito dynamics elaborates on ideas from [4]. A similar coupled model has been previously fitted to data in [5]. Our model can be written as a system of ODEs as follows:

$$\frac{dS_1}{dt} = B - \beta\, S_1 + \gamma_1 S_2 - \mu\, S_1 + (\rho_1 + k_1)\, I_1 \tag{1}$$

$$\begin{aligned}
\frac{dE_1^{(1)}}{dt} &= \beta\, S_1 - \mu\, E_1^{(1)} - \sigma\, n_H\, E_1^{(1)} \qquad (2)\\
\frac{dE_2^{(1)}}{dt} &= \sigma\, n_H\, E_1^{(1)} - \mu\, E_2^{(1)} - \sigma\, n_H\, E_2^{(1)}\\
&\cdots\\
\frac{dE_i^{(1)}}{dt} &= \sigma\, n_H\, E_{i-1}^{(1)} - \mu\, E_i^{(1)} - \sigma\, n_H\, E_i^{(1)}\\
&\cdots\\
\frac{dE_{n_H}^{(1)}}{dt} &= \sigma\, n_H\, E_{n_H-1}^{(1)} - \mu\, E_{n_H}^{(1)} - \sigma\, n_H\, E_{n_H}^{(1)}
\end{aligned}$$

$$\begin{aligned}
\frac{dI_1}{dt} &= \sigma\, n_H\, E_{n_H}^{(1)} + -\theta_1\, I_1 - (\rho_1 + k_1)\, I_1 - \mu\, I_1 \qquad (3)\\
\frac{dI_2}{dt} &= \theta_1\, I_1 - k_2\, I_2 - \mu\, I_2 + \sigma\, n_H\, E_{n_H}^{(2)} \qquad (4)\\
\frac{dS_2}{dt} &= -\gamma_1\, S_2 + k_2\, I_2 - \mu\, S_2 - \beta\, S_2 \qquad (5)
\end{aligned}$$

$$
\begin{aligned}
\frac{dE_1^{(2)}}{dt} &= \beta\, S_2 - \mu\, E_1^{(2)} - \sigma\, n_H\, E_1^{(2)} \qquad (6)\\
\frac{dE_2^{(2)}}{dt} &= \sigma\, n_H\, E_1^{(2)} - \mu\, E_2^{(2)} - \sigma\, n_H\, E_2^{(2)}\\
&\cdots\\
\frac{dE_i^{(2)}}{dt} &= \sigma\, n_H\, E_{i-1}^{(2)} - \mu\, E_i^{(2)} - \sigma\, n_H\, E_i^{(2)}\\
&\cdots\\
\frac{dE_{n_H}^{(2)}}{dt} &= \sigma\, n_H\, E_{n_H-1}^{(2)} - \mu\, E_{n_H}^{(2)} - \sigma\, n_H\, E_{n_H}^{(2)}
\end{aligned}
$$

$$
\begin{aligned}
\frac{dL}{dt} &= F\, a\, M \left(\frac{K-L}{K}\right) - \mu_L\, L - d_L\, L \qquad (7)\\
\frac{dX}{dt} &= -\left(c_1\, a\, \frac{I_1}{H} + c_2\, a\, \frac{I_2}{H}\right) X - \mu_M\, X + d_L\, L \qquad (8)
\end{aligned}
$$

$$
\begin{aligned}
\frac{dV_1}{dt} &= \left(c_1\, a\, \frac{I_1}{H} + c_2\, a\, \frac{I_2}{H}\right) X - \sigma_P n_P\, V_1 - \mu_M\, V_1 \qquad (9)\\
\frac{dV_2}{dt} &= \sigma_P\, n_P\, V_1 - \sigma_P n_P\, V_2 - \mu_M\, V_2\\
&\cdots\\
\frac{dV_i}{dt} &= \sigma_P\, n_H\, V_{i-1} - \sigma_P n_P\, V_i - \mu_M\, V_i\\
&\cdots\\
\frac{dV_{n_H}}{dt} &= \sigma_P\, n_P\, V_{n_P-1} - \sigma_P n_P\, V_{n_P} - \mu_M\, V_{n_P}
\end{aligned}
$$

$$
\begin{aligned}
\frac{dW}{dt} &= \sigma_P n_M\, V_{n_P} - \mu_M\, W \qquad (10)\\
\frac{dK}{dt} &= k_A P - k_E K \qquad (11)
\end{aligned}
$$

Eqs 1 to 6 describe the dynamics of the disease in the human population. Eqs 6 to 10 represent the population and disease dynamics of the vector. The last equation (11) describes how rainfall ($P$) controls the carrying capacity of mosquito larvae, $K$, through a conversion factor, $k_A$, and a decaying rate, $k_E$.

The above system of equations is completed by the expression for the force of infection or transmission rate:

$$\beta = b\, a\, \frac{W}{H} + \beta_e \qquad (12)$$

which includes both an external and an internal component, and introduces two new parameters: the external force of infection, $\beta_e$, and the probability of developing malaria after receiving an infectious bite, $b$.

Unlike the model in [3], here we have considered additional classes for the exposed but not yet infectious individuals We have divided both these human exposed $E$ class and the mosquito exposed $V$ class into $n_H$ and $n_V$ latent classes, respectively. This results in a gamma distribution for the two corresponding incubation times: namely, the time it takes humans to present malaria symptoms after an infectious bite, and the so-called sporogony time for the development of *Plasmodium* within the mosquito. This prescription was introduced in disease models by [6] to allow for a more flexible and realistic distribution than the exponential one. The resulting means of these incubation times are given by $1/\sigma_H$ and $1/\sigma_V$, respectively, whereas their variances decrease with the number of latent classes ($n_H$ and $n_V$).

The replenishment of susceptibles through immigration or births ($B$) and individual losses due mortality or more generally, population turnover ($\mu$), are considered to balance each other so that the total population $H$ is maintained constant. Specifically, in all our simulations, $B = \mu\, H$, where $H$ is the total human population.

Most model parameters have been described in the "Methods" section of the main text. For estimation purposes, model parameters can be divided into three different types. A first set of parameters was fixed to representative values from the literature and left unchanged across searches and simulations (Table 1). A second group of parameters (Table 2) was fitted to the malaria clinical cases observed during the period from 1970 to 1985 using an evolutionary algorithm described in [5]. Finally, a third group of parameters is known to vary as a function of rainfall and temperature (specifically, larval development rate ($d_L$), *Plasmodium* development rate ($\sigma_P$), mosquito mortality rates ($\delta_M$, $\delta_L$) and feeding rate ($a$)). These functional relationships or empirical response curves were obtained or derived from the literature.

Some parameters were typically poorly identified, with flat distributions within their interval of plausible values. For these parameters, the data does not provide additional information and solutions from the search span

| **Model Parameter** | **Symbol** | Value |
|---|---|---|
| H: Human population | $H$ | 50000 |
| H: Human turn-over rate | $\mu$ | 1/20 year$^{-1}$ |
| M: Mosquito fecundity factor | $F$ | 66 |
| T: Maximum temperature difference | $\Delta T$ | $5^{oC}$ |

Table 1: Malaria model parameters:
These parameters were maintained constant across simulations, searches, and models. H: HUMAN, M: MOSQUITO, L: LARVA, T: TEMPERATURE

this whole interval almost uniformly. Other parameter estimates were well determined. For instance, the rate $k_2$ that determines the average duration of the infectious period in the absence of drug treatment was 6.6 months with a confidence interval between 4.5 and 10.8 months. The number of exposed classes for infected humans tended to be small, resulting in a high variance of the time it takes to develop symptoms. This also holds for sporogony times, which are quite long at those altitudes (about 28 days at $20^o$C on average), but also show a high standard deviation (about 9 days). The data is also consistent with a low detection probability $\chi$, between 3% and 28% at most in that period (70-85), implying a high degree of primary infections that were either asymptomatic or undetected.

Although our model (for two immunity levels) effectively implements that of [3] by adding the coupling to a mosquito model, an interpretation difference arises in one of the parameters. Because we are fitting the model to data, we interpret the primary infected $I_1$ class as composed of individuals that show clinical symptoms. This implies that the transition from $I_1$ to $I_2$ is governed by the natural rate at which clinical symptoms disappear. By contrast, the rate $\theta_1$ in [3] represents the transition from a low immunity class $I_1$ to a high immunity class $I_2$. Their intention is to mimic the process of immunity acquisition by which adults acquire immunity with age, with children predominantly in the first class. According to these contrasting interpretations, we found $\theta_1$ values consistent with average transition times from $I_1$ to $I_2$ of the order of days (see values of the parameter $\theta_1$ in Table 2), while [3] prescribe values for $\theta_1$ corresponding to transition times of the order of years.

| Model Parameter | Symbol | Average | Confidence Interval |
|---|---|---|---|
| H: Loss of immunity rate ($S_2 \to S_1$) | $\gamma_1$ | 0.0333 | (0.0001, 0.1) |
| H: Recovery rate ($I_2 \to S_2$) | $k_2$ | 0.00469 | (0.00302, 0.00731) |
| H: Exposed number | $n_H$ | 2.22 | ( 1, 15) |
| H: Exposed rate | $\sigma$ | 0.0486 | (0.0476, 0.0504) |
| H: External force of infection | $\beta_e$ | 3.49e-05 | (8.57e-06, 9.19e-05) |
| H: Recovery rate ($I_1 \to S_1$) | $(\rho_1 + k_1)$ | 0.526 | (0.001, 1) |
| H: Recovery rate ($I_1 \to I_2$) | $\theta_1$ | 0.535 | ( 0.2, 1) |
| M: Infectivity probability ($M \to H$) | $b$ | 0.691 | ( 0.5, 0.998) |
| M: Infectivity probability ($I_1 \to M$) | $c_1$ | 0.192 | ( 0.1, 0.757) |
| M: Infectivity probability ($I_2 \to M$) | $c_2$ | 0.421 | (0.101, 0.973) |
| L: Additional death rate | $\delta_P$ | 0.0302 | ( 0.01, 0.307) |
| L: Death factor due to rain peaks | $\delta_R$ | 0.00791 | (1.24e-05, 0.129) |
| R: Carrying capacity conversion factor | $K_A$ | 922 | ( 90.8, 2.9e+03) |
| R: Carrying capacity loss rate | $K_E$ | 0.202 | (0.0786, 0.3) |
| P: Exposed number | $n_V$ | 9.85 | ( 1, 25) |
| T: $x$, $[T_e = T_o + (1 - x)(T_i - T_o)]$ | $x$ | 0.0101 | (1.28e-06, 0.0512) |
| H: Initial fraction of susceptible | $y_S[0]$ | 0.976 | ( 0.95, 1) |
| H: Initial fraction of exposed | $y_{E_1}[0]$ | 0.00424 | (0.000924, 0.0112) |
| H: Initial fraction of infectious | $y_I[0]$ | 0.00606 | (3.82e-19, 0.0194) |
| H: Initial fraction of cases | $y_C[0]$ | 0.00298 | (8.67e-21, 0.01) |
| H: Initial fraction of exposed | $y_{E_2}[0]$ | 0.00955 | (4e-05, 0.02) |
| H: Detection probability ($I_1 \to C$) | $\chi$ | 0.0974 | (0.028, 0.282) |

Table 2: Malaria model parameters:
Parameter averages and confidence intervals over the family of solutions that yield comparable fittings of our data (within 2 units of negative loglikelihood from the the best parameter set found). In addition to the dynamic parameters (see Eq. 1-11), the detection probability $\chi$ is the probability of a primary infected individual being detected and reported in the hospital, i.e., effectively counted as a malaria clinical case. H: human, M: Mosquito, L: Larva, R: Rain, P: *Plasmodium*, T: Temperature, $T_e$: Effective temperature, $(T_i - T_o) = 5^{oC}$, the maximum possible difference between indoors and outdoors temperature [7, 8].) Rates were always in units of $day^{-1}$ unless otherwise stated.

For further details on model implementation and parameter estimation see [5], and references therein.

## Trade-off between the drug-resistant and wild type strains

A *Plasmodium* drug-resistant strain is potentially favored in an environment where most of the infected population receives drug treatment. However, the evolution of drug-resistance involves a cost. Drug-resistant strains are not sensitive to drugs, but they are considered to show a concomitant shortening of the duration of infection (an increase in their natural clearance rates).

In our model, there are two clearance rates, for the transitions from $I_1$ to $S_1$ and $I_2$ to $S_2$, respectively. The first rate is enhanced by drug treatment, so it can be seen as the sum of a natural clearance rate $k_1$ plus a drug clearance rate $\rho_1$ (see Table 2). The second one is just a natural clearance rate $k_2$, because individuals in class $I_2$ do not receive any treatment. In our model (see main text), we need to distinguish these rates for the resistant parasite, $k_{1,r}$ and $k_{2,r}$, from those for the wild-type strain, $k_{1,w}$ and $k_{2,w}$.

In this analysis, the trade-off is defined as follows:

$$k_{1,r} = (1+x)(1-e)\,\rho \tag{13}$$

$$k_{2,r} = (1+x)\,k_{2,w} \tag{14}$$

where $\rho = k_{1,w} + \rho_1$.

We can then define a parameter measuring drug efficiency as $e = \rho_1/\rho$, corresponding to the fraction of the total rate which is treatment-induced. With this definition, we can re-write the trade-off simply as:

$$k_{1,r} = (1+x)\,k_{1,w} \tag{15}$$

$$k_{2,r} = (1+x)\,k_{2,w} \tag{16}$$

In sum, the cost of the resistant strain is parametrized as a fractional increase $x$ of its natural clearance rates over those of the wild-type, while the efficiency of the drug, which confers an advantage to the resistant strain, is parametrized by the $e$ parameter.

In figure SII-1 we have analyzed, for different values of $x$ and $e$, when a resistant strain is potentially favored, by using the criterion deduced in the

main text (Eqts. 3 and 4). A particular numerical integration of the ODE system given by Eqts. (1)-(11) represents the temporal evolution of the disease under wild-type infection. At each point in time, we can determine whether the resistant strain would be able to invade and establish, either from immigration from outside the system or *de novo* evolution. Because we have a family of solutions that provide comparable fits to our data, we have represented in the $y$ axis of figure SII-1, the fraction of these solutions that would allow for the invasion of a resistant strain at a given time. The main pattern is an overall decline in this fraction from the 1970s and 1980s to the 1990s, implying a decreasing ability of the resistant strain to invade, even though the actual value of this quantity depends strongly on $x$ and $e$ as expected.

In figure SII-2 we illustrate for given values of the fitness cost and treatment levels, how the difference between reproduction numbers of the resistant and wild type strains ($R_r - R_w$) changes from the 1970s to the 1990s. We plot normalized histograms of the difference for 1970-85 (in black) and 1985-2002 (in gray) (see caption for details).

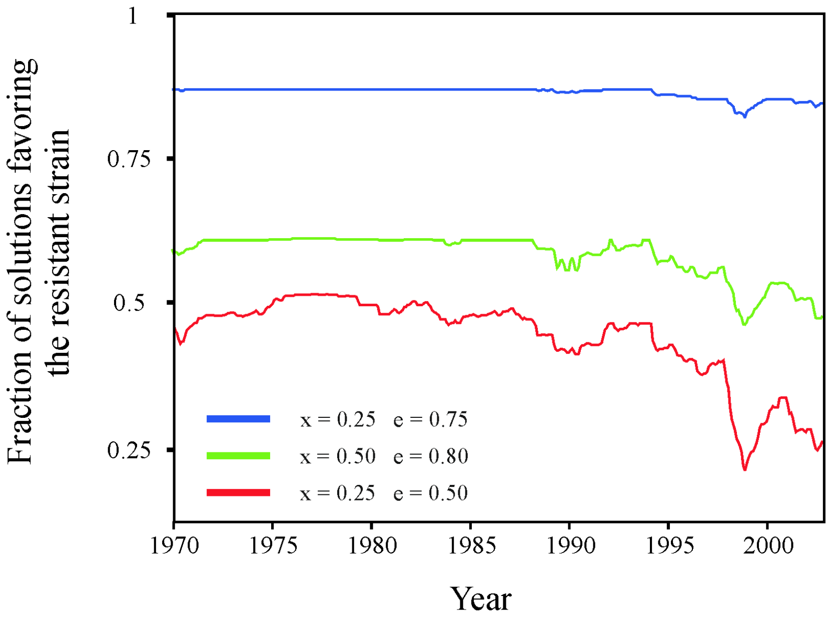


Figure SII-1: Spread of drug resistance. As drug efficiency $e$ decreases, a smaller fraction of solutions in the fitted ensemble favor the resistant strain (compare red and blue line). As the cost of resistance $x$ increases, the resistance strain is also less favored (green line). However, all curves exhibit a declining tendency for the ability of the resistant strain to invade.

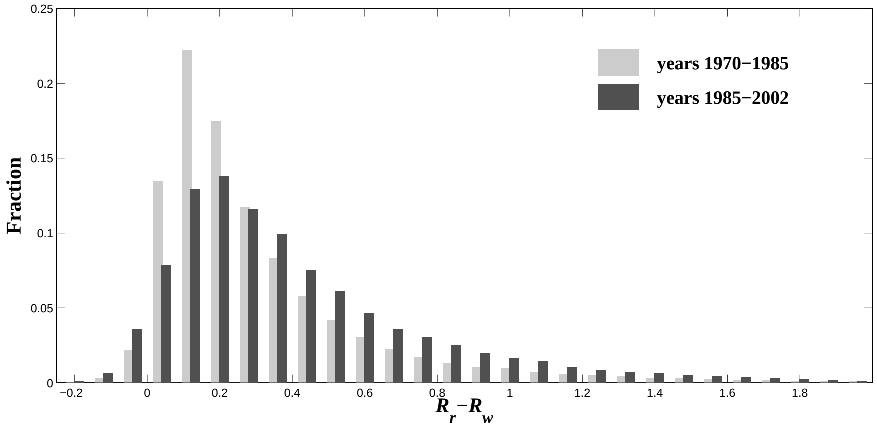


Figure SII-2: Histograms of monthly values of the difference between reproduction numbers of the resistant and wild type strains ($R_r - R_w$) for the first (1970-85, gray) and second (1985-2002, black) period under study. Monthly values are calculated through numerical integration of the system (see Eq 1-11) under the observed temperature across all parameter sets that reasonably fit monthly cases during the initial period 70-85. Reasonable fits are defined as those yielding likelihoods within 2 units of loglikelihoods from the one obtained with the best parameter set. Values of drug efficiency and resistance cost are 0.75 and 0.25, respectively, and are kept constant through all numerical integrations.

## References


[1] M. Pascual, A. Dobson, and M. J. Bouma. Underestimating malaria risk under variable temperatures. *PNAS*, 106(33):13645–13646, 2009.

[2] M. Pascual, B. Cazelles, M. J. Bouma, L. F. Chaves, and K. Koelle. Shifting patterns: malaria dynamics and rainfall variability in an African highland. *PRSLB*, 275 (1631):123–132, 2008.

[3] E. Y. Klein, D. L. Smith, M. F. Boni, and R. Laxminarayan. Clinically immune hosts as a refuge for drug-sensitive malaria parasites. *Malaria Journal*, 7:67–, 2008.

[4] J. Ahumada and A. P. Dobson. In review for *Journal of Animal Ecology*, 2010.

[5] D. Alonso, M. Bouma, and M. Pascual. Epidemic malaria and warmer temperatures in recent decades in an East African highland. *Under revision for PRSLB*, 2010.

[6] A. L. Lloyd. Realistic distributions of infectious periods in epidemic models: changing patterns of persistence and dynamics. *Theoretical Population Biology*, 60:59–71, 2001.

[7] Y. A. Afrane, B. W. Lawson, A. K. Githeko, and G. Yan. Effects of microclimatic changes caused by land use and land cover on duration of gonotrophic cycle of *anopheles gambiae* in Western Kenya highlands. *Journal of Medical Entomology*, 42:974–980, 2005.

[8] G. D. Shanks, S. I. Hay, J. A. Omumbo, and R. W. Snow. Malaria in Kenya's Western highlands. *Emerging Infectious Diseases*, 11:1425–1432, 2005.
