# Supplementary material for: Transmission Intensity and Drug Resistance in Malaria Population Dynamics: Implications for Climate Change
Source: PLoS One. 2010 Oct 26;5(10):e13588. doi: 10.1371/journal.pone.0013588 (PMC2965653; doi:10.1371/journal.pone.0013588)
Supplement: Supporting Information S3 — Relative effectively of the resistant parasite over the wild-type. (1.37 MB PDF) [file pone.0013588.s003.pdf]

Transmission intensity and drug resistance in malaria population dynamics: implications for climate change

Yael Artzy-Randrup, David Alonso & Mercedes Pascual

Supporting Information 3

In the context of the question on the role of climate change *vs.* drug resistance in areas of epidemic malaria, for which we considered a time series of monthly confirmed malaria cases from a hospital record in a tea plantation in Kericho in the highlands of Kenya, it is useful to describe how the effective basic reproductive numbers (R_w and R_r) of the two parasite types vary with vectorial capacity and with changes in the epidemiological structure of the population. We have focused earlier on whether the difference between these two is positive or negative, as an indicator for which of the two parasite types will outcompete the other, and we now focus on the rate of change of each of these as transmission intensity increases. Figure S3-1a illustrates their typical variation, which includes two noticeable regimes: ***i***) at low levels of vectorial capacity, both R_w (in blue) and R_r (in red) rapidly increase monotonically and their difference (in green) increases as well, and ***ii***) after a certain point, the changes in R_w and R_r become mild although vectorial capacity continues to increase. The increase in the difference between R_w and R_r in regime ***i***, points to an increasing effectively of the resistant parasite to spread and dominant over the wild type. Note that the turnover from regime ***i***) to ***ii***) is strongly

associated with the turnover point at which the accumulation of symptomatic infections cases in the first immunity class (I_1) reaches its maximum (see Figure S3-1 a and b).

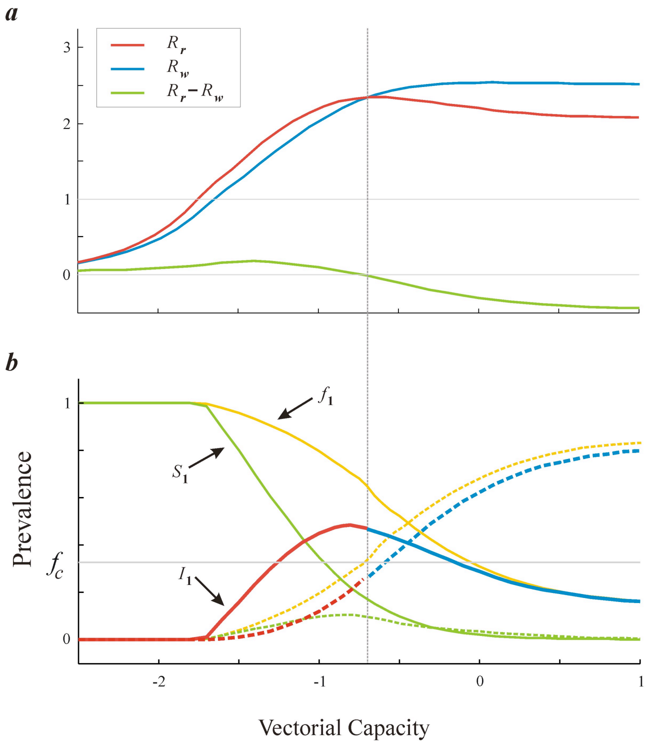

Figure S3-1**:** Simulation results of a two-class model (the x-axis is given in log scale). Fitness cost on the resistant parasite is set at ψ =0.6, and other parameters are as defined in table 2 of the Materials and Methods section. a) The effective reproductive numbers, R_w (blue) and R_r (red), in all plots are calculated according to results obtained when simulations were run in the absence resistance. These values portray the possibility of the resistant parasite to invade a population of all wild type, such that when $R_w < R_r$ and $R_r > 1$ the resistant parasite will invade and spread and otherwise it will not. The difference, $R_r - R_w$ is marked in green. b) Frequency distributions of the different classes when both resistant and will type are present in the system for the same parameters as in a). Solid lines represent immunity class 1 and dashed lines

immunity class 2. In green are the fraction of susceptibles, in yellow are the sum in the fraction of infected and susceptibles, in red are the fraction of infected with resistant and in blue are the fraction of infected with wild-type. The grey horizontal line marking f_c represents the threshold level for the second immunity class (f_2 , dashed yellow line), when resistance can invade and outcompetes the wild type (see Materials and Methods, Thresholds in a two class model). It is at this point that the difference $R_r - R_w < 0$ becomes negative, as seen in figure a) and demonstrated by the grey vertical line. Note the strong association between the rapid increase in the effective reproductive ratios and the accumulation of infected hosts in the first immunity class. Similar results are obtained for higher numbers of immunity classes.
