# Supplementary material for: Transmission Intensity and Drug Resistance in Malaria Population Dynamics: Implications for Climate Change
Source: PLoS One. 2010 Oct 26;5(10):e13588. doi: 10.1371/journal.pone.0013588 (PMC2965653; doi:10.1371/journal.pone.0013588)
Supplement: Supporting Information S4 — A revised model. (0.56 MB PDF) [file pone.0013588.s004.pdf]

Transmission intensity and drug resistance in malaria population dynamics: implications for climate change

Yael Artzy-Randrup, David Alonso & Mercedes Pascual

Supporting Information 4

We extend our model to include fitness costs on the resistant parasite in several stages of the life cycle. We distinguish here not only between the transmission probabilities of the different immunity classes (as we did earlier), but also between the resistant and the wild type. Thus, $c_{w,i}$ denotes the transmission probability of hosts infected with wild type in immunity class *i*, and $c_{r,i}$ denotes the transmission probability of hosts infected with the resistant parasite in immunity class *i*.

$$\text{class} \quad i=1: \quad \begin{cases} \dot{S}_1 = B - S_1\left(\beta\sum_{j=1}^{n}\left(c_{w,j}I_{w,j}+c_{r,j}I_{r,j}\right)+\mu\right)+I_{w,1}\left(\rho_1+\kappa_{w,1}\right)+I_{r,1}\kappa_{r,1}+\gamma_1 S_2 \\ \dot{I}_{w,1} = S_1\beta\sum_{j=1}^{n}c_{w,j}I_{w,j} - I_{w,1}\left(\rho_1+\kappa_{w,1}+\mu+\mu_{w,1}\right)-I_{w,1}\theta_1 \\ \dot{I}_{r,1} = S_1\beta\sum_{j=1}^{n}c_{r,j}I_{r,j} - I_{r,1}\left(\kappa_{r,1}+\mu+\mu_{r,1}\right)-I_{r,1}\theta_1 \end{cases}$$

$$\text{classes} \quad 1<i<n: \quad \begin{cases} \dot{S}_i = -S_i\left(\beta\sum_{j=1}^{n}\left(c_{w,j}I_{w,j}+c_{r,j}I_{r,j}\right)+\mu+\gamma_{i-1}\right)+I_{w,i}\left(\rho_i+\kappa_{w,i}\right)+I_{r,i}\kappa_{r,i}+\gamma_i S_{i+1} \\ \dot{I}_{w,i} = S_i\beta\sum_{j=1}^{n}c_{w,j}I_{w,k} - I_{w,i}\left(\rho_i+\kappa_{w,i}+\mu+\mu_{w,i}\right)-I_{w,i}\theta_i+I_{w,i-1}\theta_{i-1} \\ \dot{I}_{r,i} = S_i\beta\sum_{j=1}^{n}c_{r,j}I_{r,j} - I_{r,i}\left(\kappa_{r,i}+\mu+\mu_{r,i}\right)-I_{r,i}\theta_i+I_{r,i-1}\theta_{i-1} \end{cases}$$

$$\text{class} \quad i=n: \quad \begin{cases} \dot{S}_n = -S_n\left(\beta\sum_{j=1}^{n}\left(c_{w,j}I_{w,j}+c_{r,j}I_{r,j}\right)+\mu+\gamma_{n-1}\right)+I_{w,n}\left(\rho_n+\kappa_{w,n}\right)+I_{r,n}\kappa_{r,n} \\ \dot{I}_{w_n} = S_n\beta\sum_{j=1}^{n}c_{w,j}I_{w,j} - I_{w,n}\left(\rho_n+\kappa_{w,n}+\mu+\mu_{w,n}\right)+I_{w,n-1}\theta_{n-1} \\ \dot{I}_{r_n} = S_n\beta\sum_{j=1}^{n}c_{r,j}I_{r,j} - I_{r,n}\left(\kappa_{r,n}+\mu+\mu_{r,n}\right)+I_{r,n-1}\theta_{n-1} \end{cases}$$

Our assumptions are:

1) The change in the transmission probabilities from immunity class to immunity class stays at the same ratio for both resistant and wild type. Thus, if there is no cost of resistance, obviously, $c_{w,3}/c_{w,2} = c_{r,3}/c_{r,2}$ because $\forall i$: $c_{w,i} = c_{r,i}$. If there is cost of resistance, we assume that the change between immunity classes is constant: $c_{w,3}/c_{w,2} = c_{r,3}/c_{r,2}$. From here we find that $\alpha = c_{w,3}/c_{r,3} = c_{w,2}/c_{r,2} = c_{w,1}/c_{r,1}$.

2) The treatment level does not increase as clinical immunity is gained: $\rho_1 \geq \rho_2 \geq \rho_3$

We use the following notations to define the conditions for the valley phenomenon:

$$A_i = \rho_i + \kappa_{w,i}, \quad B_i = \kappa_{r,i}, \quad U = \mu + \mu_i, \quad x_i = \theta_i\left(c_{w,i+1}/c_{w,i}\right) = \theta_i\left(c_{r,i+1}/c_{r,i}\right), \quad y_i = \theta_i + U$$

Note the following inequality, which will be useful in several stages:

$$when \quad Y_1 > Y_2 > 0 \ \ and \ \ x > 0:$$

$$\frac{Y_1}{Y_1+x} > \frac{Y_2}{Y_2+x} \quad \Rightarrow \quad \left(\frac{Y_1}{Y_1+x}\right)\left(\frac{Y_2+x}{Y_2}\right) > 1 \quad \Rightarrow \quad \left(\frac{Y_1}{Y_2}\right)\left(\frac{Y_2+x}{Y_1+x}\right) > 1 \quad \Rightarrow \quad \left(\frac{Y_1+x}{Y_2+x}\right)\left(\frac{Y_2}{Y_1}\right) < 1$$

The conditions for the valley phenomenon in the three-class model are (in red):

$$III: \left(\frac{B_3+U}{A_3+U}\right)\underbrace{\left(\frac{c_{w,3}}{c_{r,3}}\right)}_{\geq 1} < 1 \quad \Rightarrow \quad \left\{ if \ \ \alpha \geq 1, \ we \ must \ have \ B_3 < A_3 \right.$$

$$II: \underbrace{\left(\frac{A_3+U+x_2}{B_3+U+x_2}\right)\left(\frac{B_3+U}{A_3+U}\right)}_{if\ B_3<A_3,\ this\ is\ <1}\underbrace{\left(\frac{B_2+y_2}{A_2+y_2}\right)\underbrace{\left(\frac{c_{w,2}}{c_{r,2}}\right)}_{\geq 1}}_{we\ must\ have\ this\ >1} > 1 \quad \Rightarrow \quad \begin{cases} if \ \ \alpha = 1, \ we \ must \ have \ B_2 >> A_2 \\ if \ \ \alpha > 1, \ it \ may \ be \ posible \ to \ have \ B_2 < A_2 \end{cases}$$

$$I: \left(\frac{B_1+y_1}{A_1+y_1}\right)\underbrace{\frac{\left(1+\frac{x_1}{A_2+y_2}\left(1+\frac{x_2}{A_3+U}\right)\right)}{\left(1+\frac{x_1}{B_2+y_2}\left(1+\frac{x_2}{B_3+U}\right)\right)}}_{\substack{if\ \ \alpha=1\ this\ is\ >1 \\ if\ \ \alpha>1\ this\ could\ be\ <1}}\underbrace{\left(\frac{c_{w,1}}{c_{r,1}}\right)}_{\geq 1} < 1 \quad \Rightarrow \quad \begin{cases} if \ \ \alpha = 1, \ we \ must \ have \ B_1 << A_1 \\ if \ \ \alpha > 1 \ it \ may \ be \ posible \ to \ have \ B_1 > A_1 \end{cases}$$

CASE STUDY I

Studying the case where $B_3 << A_3 \; and \; B_2 >> A_2$: (as, for example, was necessary when $\forall i: \; c_{w,i} = c_{r,i}$)

$$\kappa_{r,3} < \rho_3 + \kappa_{w,3} < \underbrace{\rho_2 + \kappa_{w,3} \le \rho_2 + \kappa_{w,2}}_{assum \; \kappa_{w,3} \le \kappa_{w,2}} < \kappa_{r,2} \quad \Rightarrow \quad even\ if\ we\ assume\ \kappa_{w,3} = \kappa_{w,2},\ we\ must\ have\ \kappa_{r,3} << \kappa_{r,2}$$

Thus, either the clearance rate decreases from class to class in the wild type, leading to a decrease in the clearance rate of the resistant type. Or, alternatively, if clearance rate of the wild type does not decrease with gain of immunity, there must be a cost of resistance on the resistant parasite that clears it more slowly in the higher immunity classes, and note that in this case, this cost of resistance **cannot be linear** (i.e., $\kappa_{w,3} = \kappa_{w,2} \; and \; \kappa_{r,3} << \kappa_{r,2}$).

CASE STUDY II

Now assume that $\forall i: \; c_{w,i} > c_{r,i}$ such that there may be a fitness cost on the resistant parasite in the probability of transmission:

$$we\ need: \quad \left(\frac{B_3 + U}{A_3 + U}\right)\alpha < 1 \;\; and \;\; \left(\frac{B_2 + y_2}{A_2 + y_2}\right)\alpha > 1 \quad \Rightarrow \quad \left(\frac{B_3 + U}{A_3 + U}\right) < \frac{1}{\alpha} < \left(\frac{B_2 + y_2}{A_2 + y_2}\right)$$

$$i)\ \ for\ \left(\frac{B_3 + U}{A_3 + U}\right)\alpha < 1 \;\; we\ must\ have\ B_3 < A_3 \;\; (necessary\ but\ not\ sufficient\ condition\ for\ III)$$

$note: \; \delta_{AB,3} = A_3 - B_3 > 0, \;\; thus:$

$$\left(\frac{B_3 + U}{B_3 + \delta_{AB,3} + U}\right) < \frac{1}{\alpha} \quad \Leftrightarrow \quad (B_3 + U)(\alpha - 1) < \delta_{AB,3}$$

we will return to this later in subsection iv

$$ii)\ \ for\ \left(\frac{B_3+U}{A_3+U}\right)<\left(\frac{B_2+y_2}{A_2+y_2}\right)\qquad we\ study\ \frac{(A_3+U)(B_2+U+\theta_2)}{(B_3+U)(A_2+U+\theta_2)}>1$$

$$we\ \ note\ \ (A_2-A_3)=\varepsilon_A>0,\qquad because:\ \ A_3=\rho_3+\kappa_{w,3}<\underbrace{\rho_2+\kappa_{w,3}<\rho_2+\kappa_{w,2}}_{assum\ \kappa_{w,3}\le\kappa_{w,2}}=A_2$$

$$we\ \ note\ \ (B_2-B_3)=\varepsilon_B\ge 0,\qquad when\ \ \kappa_{r,3}\le\kappa_{r,2}$$

$$we\ \ need:$$

$$1<\frac{(A_3+U)(B_3+\varepsilon_B+U+\theta_2)}{(A_3+U+\theta_2+\varepsilon_A)(B_3+U)}<\frac{(A_3+U)(B_3+\varepsilon_B+U+\theta_2)}{(A_3+U+\theta_2)(B_3+U)}=\underbrace{\frac{(A_3+U)(B_3+U+\theta_2)}{(A_3+U+\theta_2)(B_3+U)}}_{if\ A_3>B_3\ \ this\ is\ >1}+\underbrace{\frac{\varepsilon_B(A_3+U)}{(A_3+U+\theta_2)(B_3+U)}}_{>0}$$

$$thus\ \ A_3\ge B_3\ \ is\ a\ sufficient\ condition\ for\ \left(\frac{B_3+U}{A_3+U}\right)<\left(\frac{B_2+y_2}{A_2+y_2}\right)\ \ (both\ for\ \kappa_{r,3}=\kappa_{r,2}\ \ as\ well\ as\ \kappa_{r,3}<\kappa_{r,2})$$

$$recall\ that\ \ from\ \ condition\ \ III\ \ we\ must\ have\ B_3<A_3$$

$$iii)\ \ for\ \left(\frac{B_2+y_2}{A_2+y_2}\right)>\frac{1}{\alpha}$$

$$**\ \ either\ B_2>A_2\ \ and\ \ togethe\ with\ B_3<A_3\ \ from\ CASE\ \ STUDY\ I\ we\ must\ have$$

$$\kappa_{r,3}<<\kappa_{r,2}\ \ \ even\ \ if\ \ \kappa_{w,3}=\kappa_{w,2}$$

$$**\ \ or\ \ B_2<A_2\ \ and\ note\ \ \delta_{AB,2}=A_2-B_2>0,\ \ thus:$$

$$\left(\frac{B_2+y_2}{B_2+\delta_{AB,2}+y_2}\right)>\frac{1}{\alpha}\qquad\Leftrightarrow\qquad(B_2+y_2)(\alpha-1)>\delta_{AB,2}\qquad\quad B_2>\frac{\delta_{AB,2}}{\alpha-1}-y_2$$

$$iv)\ \ to\ fulfill\ both\ \ ii\ \ and\ iii:$$

$$B_2>\frac{\delta_{AB,2}}{\alpha-1}-\theta_2-U\ \ \ and\ \ B_3<\frac{\delta_{AB,3}}{\alpha-1}-U$$

$$if\ \ \ B_2=B_3:\qquad\frac{\delta_{AB,2}}{\alpha-1}-\theta_2-U<B_3<\frac{\delta_{AB,3}}{\alpha-1}-U\quad\Rightarrow\quad(\alpha-1)\theta_2>\delta_{AB,2}-\delta_{AB,3}=A_2-A_3\ge 0$$

$\Rightarrow$ *if i) the difference in treatment between immnuity class 2 and immnuity class 3 is very small,*
or if ii) imunity is gained at a fast rate,
or if iii) the transmission probability of the wild type is much higher than the resistant
it may be possible to get the valley phenomenon when there is no increase in duration:
$\kappa_{r,3}=\kappa_{r,2}$ *and* $\kappa_{w,3}=\kappa_{w,2}$

Some general conclusions on the necessary conditions for the valley phenomenon:

1) We find that if there is no fitness cost on probability of transmission ($\forall i: \; c_{w,i} = c_{r,i}$), the clearance rate of the resistant parasite must decrease with gain of immunity.
This would be true both if the cost of resistance decreases the clearance rate of the resistant parasite or increases it.

2) If there is a fitness cost on transmission probability, it may be possible to find the valley phenomenon while assuming there is no reduction in clearance rate between the immunity classes (both for the resistant and the wild type).

3) An increase or a decrease in the transmission probabilities between the immunity classes plays no role on the conditions for the valley phenomenon.
